# MET Inhibition Sensitizes Rhabdomyosarcoma Cells to NOTCH Signaling Suppression

**DOI:** 10.3389/fonc.2022.835642

**Published:** 2022-04-27

**Authors:** Clara Perrone, Silvia Pomella, Matteo Cassandri, Michele Pezzella, Giuseppe Maria Milano, Marta Colletti, Cristina Cossetti, Giulia Pericoli, Angela Di Giannatale, Emmanuel de Billy, Maria Vinci, Stefania Petrini, Francesco Marampon, Concetta Quintarelli, Riccardo Taulli, Josep Roma, Soledad Gallego, Simona Camero, Paolo Mariottini, Manuela Cervelli, Roberta Maestro, Lucio Miele, Biagio De Angelis, Franco Locatelli, Rossella Rota

**Affiliations:** ^1^ Department of Hematology and Oncology, Cell and Gene Therapy, Bambino Gesù Children’s Hospital, IRCCS, Rome, Italy; ^2^ Department of Science, “Department of Excellence 2018-2022”, University of Rome “Roma Tre”, Rome, Italy; ^3^ Department of Radiotherapy, Sapienza University, Rome, Italy; ^4^ Confocal Microscopy Core Facility, Bambino Gesù Children’s Hospital, IRCCS, Rome, Italy; ^5^ Department of Translational Medical Sciences, University of Naples Federico II, Naples, Italy; ^6^ Department of Oncology, University of Torino, Torino, Italy; ^7^ Group of Translational Research in Child and Adolescent Cancer, Vall d’Hebron Research Insti-tute-Universitat Autònoma de Barcelona, Barcelona, Spain; ^8^ Department of Maternal, Infantile and Urological Sciences, Sapienza University of Rome, Rome, Italy; ^9^ Unit of Oncogenetics and Functional Oncogenomics, Centro di Riferimento Oncologico di Aviano (CRO Aviano) IRCCS, National Cancer Institute, Aviano, Italy; ^10^ Department of Genetics and Stanley S. Scott Cancer Center, Louisiana State University Health Sciences Center, New Orleans, LA, United States; ^11^ Department of Pediatrics, Sapienza University, Rome, Italy

**Keywords:** rhabdomyosarcoma, NOTCH signaling, MET, soft tissue sarcoma, drug resistance, combination therapy, targeted therapy, γ-secretase

## Abstract

Rhabdomyosarcoma (RMS) is a pediatric myogenic soft tissue sarcoma. The Fusion-Positive (FP) subtype expresses the chimeric protein PAX3-FOXO1 (P3F) while the Fusion-Negative (FN) is devoid of any gene translocation. FP-RMS and metastatic FN-RMS are often unresponsive to conventional therapy. Therefore, novel therapeutic approaches are needed to halt tumor progression. NOTCH signaling has oncogenic functions in RMS and its pharmacologic inhibition through γ-secretase inhibitors blocks tumor growth *in vitro* and *in vivo*. Here, we show that NOTCH signaling blockade resulted in the up-regulation and phosphorylation of the MET oncogene in both RH30 (FP-RMS) and RD (FN-RMS) cell lines. Pharmacologic inhibition of either NOTCH or MET signaling slowed proliferation and restrained cell survival compared to control cells partly by increasing Annexin V and CASP3/7 activation. Co-treatment with NOTCH and MET inhibitors significantly amplified these effects and enhanced PARP1 cleavage in both cell lines. Moreover, it severely hampered cell migration, colony formation, and anchorage-independent growth compared to single-agent treatments in both cell lines and significantly prevented the growth of FN-RMS cells grown as spheroids. Collectively, our results unveil the overexpression of the MET oncogene by NOTCH signaling targeting in RMS cells and show that MET pathway blockade sensitizes them to NOTCH inhibition.

## Introduction

Rhabdomyosarcoma (RMS) is the most frequent soft tissue sarcoma of childhood that includes two major histological subtypes: embryonal and alveolar (1). About 80% of alveolar RMS shows chromosomal translocations that result in the expression of the oncogenic chimeric transcription factors (TFs) PAX3-FOXO1 or PAX7-FOXO1 (Fusion-Positive, FP) (1–3). The remaining alveolar and embryonal RMSs are Fusion-Negative (FN) and show chromosomal aberrations and often RAS mutations (3–5). Despite the improvement in the response to multimodal therapy, the prognosis for FP-RMS and metastatic FN-RMS remains dismal (1, 6), suggesting that novel therapeutic approaches are needed to halt the emergence of drug resistance. As a pediatric cancer, RMS shows a low mutational burden suggesting that de-regulation of pathways driving embryonal development, rather than gene mutations, strongly contribute to the pathogenesis (4, 7). As a matter of fact, RMS cells are thought to derive from mesenchymal progenitors of the skeletal muscle lineage that, despite the expression of the master muscle TFs such as MYOD and MYOG, are unable to differentiate and proliferate indefinitely. In line with this, we and others have demonstrated the involvement of regulators of embryonal tissue differentiation in RMS pathogenesis including the Polycomb proteins EZH2 (8, 9) and BMI1 (10), the Hippo (11–13), Hedgehog [ (14); and reviewed in (15)] and NOTCH pathways (16–18).

NOTCH signaling is an evolutionarily conserved pathway that crucially regulates cell fate during embryonal development balancing cell proliferation, differentiation, and survival [reviewed in (19)]. Human cells harbor four NOTCH genes located on different chromosomes, NOTCH1-4 [reviewed in (20)]. The NOTCH paralogues are surface membrane receptors involved in cell-to-cell communication that, once bound by their specific ligands of the Delta-like (DLL1, 3, and 4) and Serrate/Jagged (JAG1 and 2) families on the membrane of neighboring cells, are subjected to sequential proteolytic cleavages. The latter is made in the transmembrane domain by the multisubunit protease γ-secretase to release a NOTCH Intracellular Domain (ICD). Then, from the cytoplasm, NOTCH (N)ICD migrates into the nucleus where it binds the DNA-binding transcription factor CSL/RBP-Jk (CBF1/RBPj/Su (H)/Lag-1) and the mastermind-like proteins (MAML1-3) displacing the co-repressor complex and allowing the transcription of canonical NOTCH target genes of the basic helix-loop-helix (bHLH) Hes (HES1-7) and Hey (HEY1, 2, L) families of transcriptional repressors [reviewed in (20)]. Moreover, NOTCH pathway also has non-canonical functions that are still under investigation (21). Notably, NOTCH functions are strictly context- and tissue-dependent and often paralogue-specific [reviewed in (20)].

NOTCH pathway functions are deregulated in different types of hematological and solid tumors and the inhibition of NOTCH signaling through inhibitors of γ-secretase or monoclonal antibodies against specific NOTCH receptors are being evaluated in clinical settings [reviewed in (20)].

The role of NOTCH signaling during myogenesis is crucial in defining skeletal muscle fate and in promoting muscle stem cell expansion and migration [reviewed in (22–27)]. We and others have recently demonstrated that NOTCH signaling components are up-regulated and the pathway hyper-activated in RMS and that its blockade through genetic silencing or γ-secretase inhibitors leads to cell differentiation and cell cycle arrest *in vitro* and halts tumor growth *in vivo* (16, 28, 29). These findings suggest potential therapeutic value for an anti-NOTCH approach in RMS. However, clinical studies showed that treatment with agents targeting single molecules can fail due to the emergency of drug resistance and co-inhibition of multiple therapeutic targets is pursed as a strategy to give a durable clinical responses [reviewed in (28–30)].

NOTCH signaling has been reported to transcriptionally block the expression of the MET proto-oncogene in breast cancer cells (31). MET is a cell membrane-spanning receptor with tyrosine kinase activity [reviewed in (32)], which is induced by PAX3 during skeletal muscle tissue development (33) and by PAX3-FOXO1 in RMS (34). Its activation by the HGF ligand is needed in the early myogenic phases for myoblasts migration and proliferation and its expression is quickly down-regulated during the late myogenesis (35–37). MET signaling is often deregulated in cancer by over-activation, gene amplification and/or mutation, and supports tumorigenesis and metastases and drug resistance [reviewed in (38, 39)].

In RMS, MET has been shown to be over-expressed, amplified and correlated to poor prognosis in patients, and MET signaling hyper-activated and oncogenic (40–44). In line, pharmacologic or genetic inhibition of MET decreases RMS cell proliferation and migration *in vitro* and tumor growth and metastasis formation *in vivo* (45–48).

Therefore, based on this, here we evaluated whether the inhibition of NOTCH signaling affects MET expression/activation in RMS. Our findings show that genetic or pharmacologic NOTCH inhibition leads to MET up-regulation and activation in RMS cell lines known to be sensitive to NOTCH depletion, regardless of the presence of the fusion protein. In agreement, co-inhibition of NOTCH and MET signaling significantly enhanced growth inhibition and cell death of RMS cells compared to single treatments. Moreover, co-treatment with NOTCH inhibitors (NOTCHi) and MET inhibitors (METi) further limited the tumorigenic properties of RMS cells *in vitro* compared to each agent alone.

Altogether, our findings show that targeting NOTCH pathway in RMS could activate and up-regulate MET with a mechanism that needs to be clarified in future studies. They also suggest that MET inhibition could be a strategy to boost the effects of NOTCH blockade in this tumor.

## Materials and Methods

### Cell Culture and Reagents

RH30 and RH4 (PAX3-FOXO1 expressing alveolar RMS, fusion positive), and RD and JR1 (embryonal RMS, fusion negative) cell lines were gifts from Dr. Peter Houghton. Several first passage aliquots of each cell line were stored in liquid nitrogen for subsequent assays. Each aliquot was passaged for a maximum of 4 months. Cell lines were authenticated by genotyping. RH30 and RH4 cells were cultured in RPMI 1640 and RD and JR1 cells in DMEM high-glucose (Invitrogen, Carlsbad, CA, USA), both supplemented with 10% FBS, 1% L-glutamine, and 1% penicillin-streptomycin. Cells were cultured at 37°C in a humidified atmosphere of 5% CO2/95% air. All cell lines were routinely tested for Mycoplasma at the beginning of culture of each aliquot and then each two months.

### Drugs

PF-03084014 (Nirogacestat, #S8018), ARQ197 (Tivantinib, #S2753), EMD-1214063 (Tepotinib, # S7067), SU11274 (#S1080) were purchased from Selleckchem (www.selleckchem.com). BMS-777607 (CT-BMS777) and Cabozantinb (CT-XL184) were purchased from ChemieTek (Indianapolis, IN, USA). GSI-XII (Z-Ile-Leu-aldehyde, HY-12465) was purchased from MedChemExpress (Monmouth Junction, NJ, USA). All compound were dissolved in DMSO to a final concentration of 10 mmol/L.

### Determination of the IC50 and Cell Proliferation Assay

RH4, RH30, RD and JR1 cells were seeded on 384-well plates in 10% FBS-containing RPMI-1640 or DMEM high-glucose media and after 24h were treated with decreasing doses of each drug (PF-03084014 (40 µM-0.195 nM), GSI-XII (40 µM-0.195 nM), ARQ-197 (3 µM-0.0015 nM), BMS-907351 (10 µM-0.005 nM), SU-11274 (40 µM-0.195 nM), BMS-777607 (40 µM-0.195 nM), EMD-1214063 (40 µM-0.195 nM)) or with DMSO. Cells were plated to achieve 20% confluence at the time of drug treatment (1.2x103/well) and monitored until 72h, when control (DMSO-treated) wells reached ~90% confluence. IC50 values were calculated 72h post treatment using the GraphPad™ Prism version 8. For proliferation experiments, were seeded on 96-well plates (3000 cells/well) and, after 24h (t0), new media containing DMSO or the drugs at the selected concentrations were added to the wells. The percentage of cell confluence was quantified under phase contrast every 24h using the Celigo Image Cytometer (Nexcelom Bioscience, Lawrence, MA, USA). Seventy-two hours post-treatment, RH30 and RD cells were stained with Calcein AM and Propidium Iodide (PI) and the percentage of Calcein- and PI-positive cells was quantified using Celigo Image Cytometer (Nexcelom Bioscience, Lawrence, MA, USA).

### siRNA Transfection

Cells were transfected with 100nM (final concentration) siRNAs against human NOTCH1 (SASI_Hs01_00052328), human NOTCH3 (SASI_ Hs01_00101285) or a non-targeting siRNA as control (SIC001) (Sigma, St Louis, MO, USA) using Oligofectamine (Invitrogen, Carlsbad, CA), according to the manufacturer’s recommendations. Twenty-four hours later, the medium was replaced with fresh complete growth medium and transfected cells were harvested at different time points.

### RNA Isolation, cDNA Synthesis and Real-Time qPCR

Total RNA was extracted using TRIzol (Invitrogen, Carlsbad, CA, USA) according to the manufacturer’s protocol. RNA concentration was quantified using a NanoDrop^®^ 1000 Spectrophotometer (ThermoFisher Scientific). Reverse transcription was performed using the Improm-II Reverse Transcription System (Promega, Madison, WI, USA). The expression levels were measured by qRT-PCR for the relative quantification of the gene expression as described (49). TaqMan gene assay (Applied Biosystems, Life Technologies, Carlsbad, CA, USA) for MET (Hs01565584_m1), NOTCH1 (Hs01062014_m1), NOTCH3 (Hs01128541_m1), HES1 (Hs00172878_m1) were used. Values were normalized according to the glyceraldehyde-3-phosphate dehydrogenase (GAPDH) mRNA (Hs99999905_m1) levels. The QuantStudio 3 Real-Time PCR System (Applied Biosystems) was used for the measurements. The expression fold change was calculated by the 2-ΔΔCt method for each of the reference genes (49).

### Western Blot

Western blotting was performed on whole-cell lysates by homogenizing cells in RI-PA lysis buffer (50 mM Tris pH 7.4, 150 mM NaCl, 1% Triton X-100, 1 mM EDTA, 1% sodium deoxycholate, 0.1% SDS), containing the protease inhibitor cocktail (Sigma, St Louis, MO, USA), NaF 1mM, Na3VO4 1mM and PMSF 1mM. Lysates were incubated on ice for 30 minutes (min) and centrifuged at 12,000xg for 20 min at 4°C. Supernatants were used as total lysates. Protein concentrations were estimated with the BCA protein assay (Pierce, Rockford, IL), according to the manufacturer’s protocol. The proteins (40 μg) were boiled in reducing SDS sample buffer (200 mM Tris–HCl pH 6.8, 40% glycerol, 20% β-mercaptoetanol, 4% sodium dodecyl sulfate, and bromophenol blue), and run on 8% SDS-polyacrylamide gels. Then, the proteins were transferred to Hybond ECL membranes (Amersham, GE HEALTHCARE BioScience Corporate Piscataway, NJ, USA). Membranes were blocked in 5% non-fat dried milk in Tris-buffered saline (TBS) for 1h and incubated overnight (ON) with the appropriate primary antibody at 4°C. After incubation, membranes were washed in TBS and incubated with the appropriate secondary antibody for 1h at room temperature (RT). Detection was performed by Pierce™ ECL Western Blotting Substrate (Thermo Scientific™) or Western Lightning ECL Pro (PerkinElmer, Waltham, MA, USA). Antibody against MET (sc-10) was from Santa Cruz Biotechnology Inc., (Santa Cruz, CA, USA); NOTCH1 (bTAN 20) was from Developmental Studies Hybridoma Bank (DSHB, University of Iowa, Iowa City, IA, USA); α-Tubulin (NB100-92249) was from Novus Biologicals (Littleton, CO, USA); β-ACTIN (A2066) was from Sigma-Aldrich (St Louis, MO, USA); NOTCH3 (PAB-10683) was from Orbigen Inc. (Orbigen, San Diego, CA, USA). Antibodies against Cleaved NOTCH1 (Val1744) #4147), Phospho-MET (Tyr1234/1235) (#3077), PARP1 (#9542), p21Cip1 (#2947); GAPDH (#5174) and all secondary antibodies were obtained from Cell Signaling Technology (Beverly, MA, USA). All antibodies were used in accordance with the manufacturer’s instructions.

### Caspase Activity Assay

Cells were seeded into 96-well black, flat bottom plates at a density of 5000 cells per well and incubated for 24h to allow cell surface adhesion. Twenty-four hours after the cell seeding, the cells were treated with DMSO, PF-03084014, and ARQ-197 for 24h. The activity of Caspase-3/7 was determined with a Caspase-Glo-3/7 assay (Promega Company, Madison, WI, USA), according to the manufacturer’s protocol, using the EnSpire^®^ Multimode Plate Reader (PerkinElmer, Waltham, MA, USA).

### Annexin V Determination

Cells were seeded into 6-well plates (120.000 cells/well) and treated with DMSO, PF-03084014, and ARQ197 for 48h. After treatment, cells were harvested and cells suspension were incubated with PE-conjugated Annexin V and 7-Aminoactinomycin D (7-AAD) in binding buffer for 15 min in the dark, using Annexin V apoptosis detection kit (BD Pharmingen, San Diego, CA, USA), according to manufacturer’s recommendations. Cells were analyzed using FACS CantoII equipped with a FACSDiva 6.1 CellQuest software (Becton Dickinson Instrument, San Josè, CA, USA).

### Immunofluorescence

RH30 and RD cells were fixed after 8 and 24 hours of either vehicle, each drug alone, or combination treatment in 4% paraformaldehyde (PFA)/PBS for 15 min at RT, permeabilized in 0.2% Triton X-100/PBS for 5 min at RT and incubated with either rabbit phospho-MET (Tyr1234/1235) (#3077 from Cell Signaling Technology, (CST) Danvers, MA, USA) and rabbit MET (sc-10 from Santa Cruz Biotechnology, Inc., Heidelberg, Germany) in 1% BSA/PBS. Alexa-488 goat α-rabbit (Invitrogen, Carlsbad, CA, USA) was used as secondary antibody. Cells were counterstained with DAPI and imaged using the Olympus microscope FV3000 with Olympus FV315S-SW image acquisition software. Images were processed by Adobe Photoshop. The intensity average of phospo-MET and MET fluorescences were calculated using FV315S-SW software, from cytometric measurements relative to total cell area in 5 digital images randomly selected and analyzed for each immunostained cellular sample. From 80 to 100 cells were counted for each sample analyzed.

### Cell Migration Assay

Cells were seeded at 100% of confluence in medium supplemented with 10% FBS on 96-well plate using Oris Cell Migration Assay Kit (#CMA1.101) (Platypus Technologies, Madison, WI, USA). The day after, stoppers were removed and cells were cultured in 2% FBS for 24h. Analysis of cell migration into the detection zone was performed after fixing and staining with Diff-Quick^®^ (Medion Diagnostic AG460.053, Switzerland). The images were taken using the Leica DMi8 with LAS X Navigator image acquisition software.

### Colony Formation Assay

Cells were seeded at the density of 103/well in 6-well plates with 2 mL of complete growth medium. Medium with drug was replaced 24h post seeding. After 14 days cells were fixed, stained with Diff-Quik^®^ (Medion Diagnostic AG460.053, Switzerland) following the manufacturer’s instructions and the colonies containing >50 cells counted.

### Soft Agar Colony Formation Assay

Cells were assayed for their capacity to form colonies in soft-agar, as described (50). Cells (104/well) were suspended in complete growth media containing 0.5% Agar (NuSieve GTG Agarose, Lonza, Rockland, ME, USA). Then, they were seeded on a layer of 1% Agar in complete growth media in 6-well plates. Medium with drug was replaced 24h post seeding and refreshed every 2 days. After 14 days the colonies were counted by microscopic inspection and images were acquired with a Leica DMi8 with LAS X Navigator image acquisition software.

### Spheroid Generation and Image Acquisition

For 3D tumor spheroids assessment cells were seeded in 100 µl of complete growth media on 96 Ultra-Low Attachment plates (ULA, #7007) (CORNING, New York, NY, USA) to obtain tumor spheroids of 300 µm diameter in 96h. After 72h of drug treatments, diameter was measured by Celigo image cytometer (Nexcelom Bioscience, Lawrence, MA, USA). Then, after 2 or 3 days of treatment, 3D tumor spheroids were stained using PI (1 mg/ml final concentration), Calcein AM (1 mM final concentration) and Hoechst (1:10000). Image capture and analysis were performed using Celigo image cytometer (Nexcelom Bioscience, Lawrence, MA, USA).

### Statistical Analysis

Results were expressed as the mean ± standard deviation (SD) calculated on three independent experiments, unless mentioned otherwise. P values were calculated using two-tailed Student’s t-test (comparison between two groups) and two-way ANOVA (multiple group comparison). Only the variations with a statistical significance (P) <0.05 were considered significant and reported on the related figure legends. Statistical analysis was performed using GraphPad Prism version 8 (GraphPad Software; www.graphpad.com).

## Results

### NOTCH Signaling Inhibition Drives MET Activation in RMS Cells

**Figure 1:**
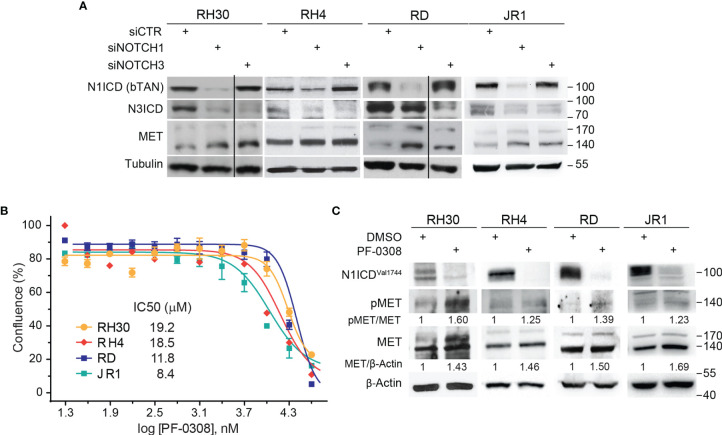
NOTCH signaling inhibition drives MET up-regulation and activation in RMS cells. **(A)** Representative (n=3 independent biological replicates) Western Blot depicting the effect of NOTCH1 and NOTCH3 silencing on MET protein levels in FP-RMS (RH30, RH4) and FN-RMS (RD, JR1) cells. Cells were harvested 48h post-transfection and the protein levels of the intracellular cleaved domain (ICD) of NOTCH1 and NOTCH3 (N1CD and N3ICD) were assessed (siCTR indicates the control siRNA sample). Migration of molecular weight markers is indicated on the right (kDa). Tubulin levels were used as loading control. **(B)** Dose-response curves of RH30, RH4, RD and JR1 cells treated with the NOTCH inhibitor PF-03084014 (PF-0308). Graph represents the mean of three independent experiment ± SEM. **(C)** Representative (n = 3 independent biological replicates) Western Blot of RH30 and RH4 cells treated with PF-03084014 (20 μM) for 48h and of RD and JR1 cells treated with PF-03084014 (10 μM) for 72h. The radiographs show total (MET) and activated/phosphorylated (pMET ^Y1234/1235^) MET protein levels. N1ICD^Val1744^ levels have been used as treatment control and β-Actin as loading control. Migration of molecular weight markers is indicated on the right (kDa).

In order to evaluate whether MET levels are modulated after NOTCH signaling inhibition, we silenced NOTCH1 and NOTCH3, the two NOTCH receptors hyper-activated in our cell tumor context (16–18), in two FP-RMS (RH30 and RH4) and two FN-RMS (RD and JR1) cell lines. As shown in **Figure 1A**, MET protein levels [reviewed in (32)] were up-regulated after 48 hours (48h) of NOTCH1 and NOTCH3 depletion in all four cell lines compared to control siRNA cells. Even if *MET* transcript levels were increased by NOTCH1 knockdown (KD) in three of four cell lines, the modulation did not reach the statistical significance (**Figure S1A**). These results suggest that MET is indirectly down-regulated by NOTCH signaling in RMS cells.

Then, we investigated whether pharmacologic inhibition of NOTCH signaling affected MET levels similarly. To this end, we used the γ-secretase inhibitor PF-03084014 (Nirogacestat), a pan-NOTCH inhibitor currently in advanced clinical development, which shows low toxicity in patients (www.clinicaltrials.gov Identifier NCT04195399) [reviewed in (20)], hereafter indicated as PF-0308. The drug affected cell proliferation of all four RMS cell lines 72h post-treatment in a µM range compared to vehicle (**Figure 1B**). MET modulation was molecularly investigated in response to PF-0308 used at 20 μM on RH30 and RH4 FP-RMS and 10 μM on RD and JR1 FN-RMS cells, respectively, throughout the study. Inhibition of NOTCH signaling by PF-0308 treatment was confirmed in four cell lines by the decrease of the levels of NOTCH1 cleaved intracellular domain (N1ICD), as revealed by the N1ICD^Val1744^-specific antibody, associated to the transcriptional down-regulation of the four canonical NOTCH target gene *HES1* (**Figure 1C** and **Figure S1B**). PF-0308 treatment mirrored the effects of NOTCH1 and NOTCH3 gene silencing up-regulating the protein levels of total MET along with those of its activated form phosphorylated at the major phosphorylation site (pMET^Tyr1234-Tyr1235^, hereafter indicated as pMET) (**Figure 1C**) in all RMS cell lines, while increased *MET* mRNA levels only in the two FN-RMS cell lines (**Figure S1C**). These results were confirmed on RH30 and RD cells using a different γ-secretase inhibitor, GSI-XII, already used in RMS (17), which also promoted MET up-regulation/phosphorylation in both cell lines (**Figures S2A, B**).

Altogether, these findings indicate that NOTCH signaling inhibition causes MET up-regulation and phosphorylation in RMS cells.

### Pharmacologic Inhibition of NOTCH Signaling Halts RMS Cells Proliferation

**Figure 2:**
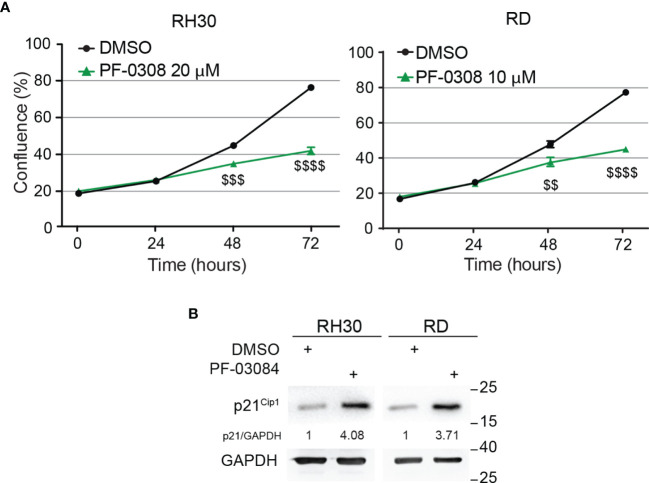
Pharmacologic inhibition of NOTCH signaling hampers RMS cells growth. **(A)** Growth-curve analysis of RH30 (left) and RD (right) cells treated with PF-03084014 (20 μM and 10 μM, respectively) for 24h, 48h and 72h. Graph represents the mean of three independent experiments ± SD, Student two-tailed T-Test. $$ P-value ≤ 0.01, $$$ P-value ≤ 0.001, $$$$ P-value ≤ 0.0001 for drug-treated *vs* vehicle-treated (DMSO) cells. **(B)** Representative radiographs showing p21^Cip1^ protein levels in RH30 and RD cells treated with PF-03084014 (20 μM for 48h and 10 μM for 72h, respectively). Migration of molecular weight markers is indicated on the right (kDa). GAPDH was used as loading control.

Then, we functionally evaluated the effects on cell growth of NOTCH signaling inhibition by PF-0308 using the chosen doses of the compound. As reported in **Figure 2A**, cell proliferation of both RD and RH30 cells was significantly reduced 48h and 72h post-treatment compared to vehicle (DMSO)-treated (control) cells. Specifically, 72h post-treatment the reduction reached about 48 ± 1.9% in RH30 and 44 ± 1.5% in RD cells (**Figure 2A**). In agreement, 72h of treatment resulted in a significant decrease in the percentage of RH4 and JR1 cells when compared to vehicle-treated cells (53 ± 0.4% and 44 ± 5.8% reduction in RH4 and JR1 cells, respectively) (**Figure S3A**). Cell proliferation was also significantly reduced in RH30 and RD cells 72h after treatment with GSI-XII ruling out off-target effects of the PF-0308 (**Figure S3B**). At the molecular level, PF-0308 treatment markedly increased the expression of the Cyclin-Dependent Kinase (CDK) inhibitor p21^Cip1^, which is known to restrain cell proliferation, in all the cell lines (**Figure 2B** and **Figure S7E**) (18, 51, 52).

These findings indicate that pharmacologic inhibition of NOTCH signaling affects the ability of RMS cells to proliferate *in vitro*.

### MET Inhibition Affects RMS Cells Proliferation

**Figure 3:**
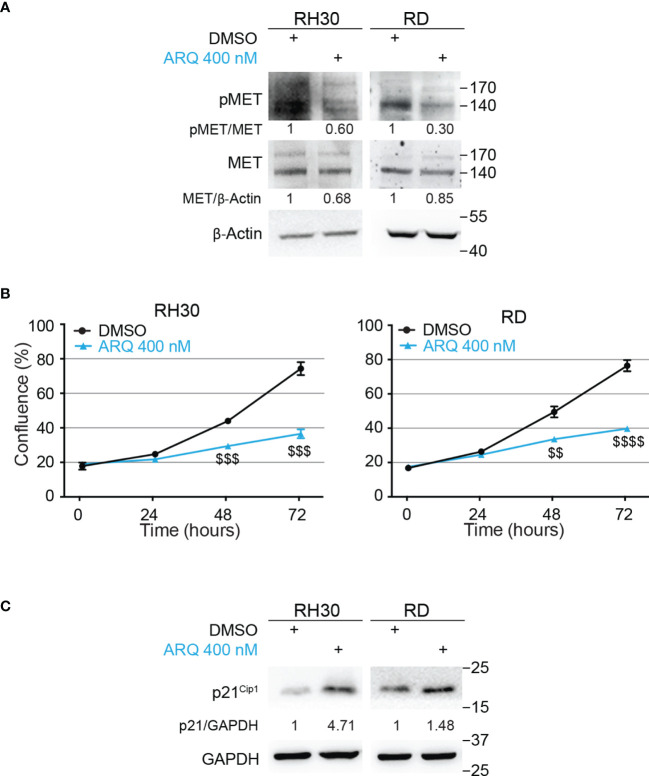
Pharmacologic inhibition of MET signaling causes RMS cells growth arrest. **(A)** Representative radiographs showing the effect of ARQ197 (ARQ 400 nM) on RH30 cells (48h) and RD cells (72h) on total and phosphorylated (pMET^Y1234/1235^) MET protein levels. Migration of molecular weight markers is indicated on the right (kDa). β-Actin was the loading control. **(B)** Growth-curves of RH30 (left) and RD (right) cells treated with ARQ197 (ARQ 400 nM) for 24h, 48h and 72h. Graphs represent the mean of three independent experiments ± SD, Student two-tailed T-Test. $$ P-value ≤ 0.01, $$$ P-value ≤ 0.001, $$$$ P-value ≤ 0.0001 for drug-treated cells *vs* vehicle-treated (DMSO) cells. **(C)** Representative radiographs showing p21^Cip1^ protein levels in RH30 and RD cells treated with 400 nM of ARQ197 for 48h and 72h, respectively. Migration of molecular weight markers is indicated on the right (kDa). GAPDH was used as loading control.

To identify a METi useful for a potential combinatorial approach with the NOTCHi, we tested, on RH30 and RD cells, five MET inhibitors with different mechanisms of action, some of which were already used in RMS cells (45–47) (**Figure S4**). ARQ-197 (Tivantinib), hereafter indicated as ARQ, was then chosen for the highest effects on cell growth at nM doses compared to vehicle-treated cells. As this MET inhibitor had never been tested on RMS cells, we evaluated whether it specifically targets MET. The decrease of phosphorylated MET levels observed in the four RMS cell lines demonstrated a specific MET targeting upon ARQ 400 nM treatment (**Figure 3A** and **Figure S7D**).

Moreover, ARQ 400 nM significantly reduced cell proliferation 72h post-treatment in RMS cell lines compared to vehicle-treated cells (54 ± 2.6%, 41 ± 1.8%, 42 ± 3.0% and 33 ± 0.4% growth reduction in RH30, RD, RH4 and JR1 cells, respectively) (**Figure 3B** and **Figures S5A, B**). It also increased p21^Cip1^ protein levels (**Figure 3C** and **Figure S7E**).

Altogether, these findings suggest that the METi ARQ reduces MET phosphorylation and significantly affects RMS cell proliferation.

### Combined Inhibition of NOTCH and MET Signaling Enhances RMS Cells Apoptosis

**Figure 4:**
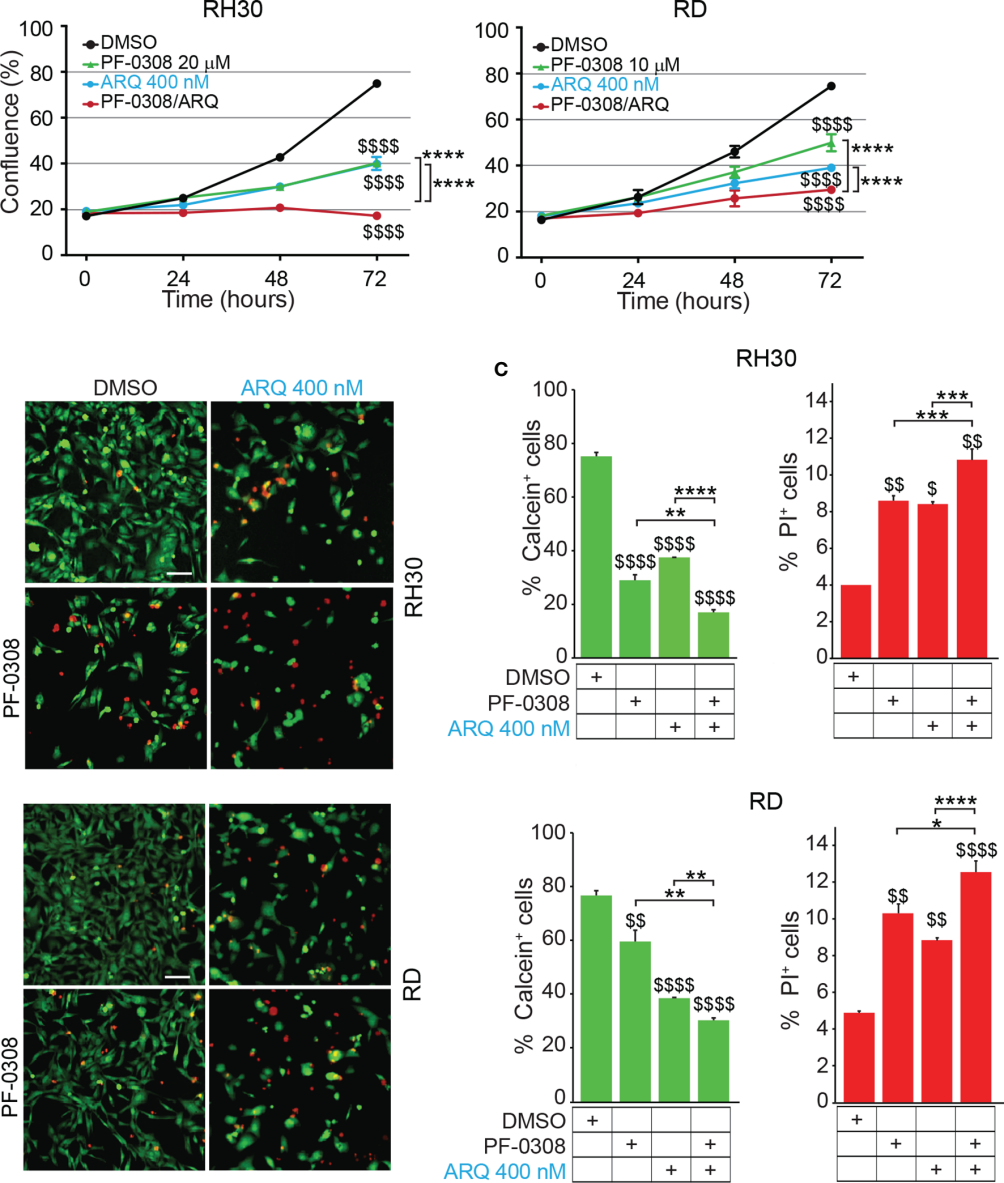
NOTCH and MET signaling co-inhibition hampers RMS cells growth and induces cell death. **(A)** Growth-curves of RH30 and RD cells treated for 24h, 48h and 72h with either 20 µM or 10 µM of PF-03084014, respectively, or 400 nM of ARQ197 or with the drug combination. Graphs represent the mean of three independent experiments ± SD, 2-way ANOVA test. $$$$ P-value ≤ 0.0001 for drug-treated *vs* vehicle-treated (DMSO) cells, and ****P-value ≤ 0.0001 for drug combination-treated cells *vs* single agents. **(B)** Representative images of Calcein AM (green: alive cells) and Propidium Iodide (PI; red: dead cells) staining of RH30 and RD cells treated for 72h with either 20 μM and 10 μM PF-03084014, respectively, or 400 nM of ARQ197 (ARQ 400 nM) or with the drug combination. Scale bars = 50 μm. **(C)** Histograms represent quantitative analysis of Calcein AM (green: alive cells) and Propidium Iodide (PI; red: dead cells) positive cells for RH30 and RD treated as in **(B)**. Graphs represent the mean of three independent experiments ± SD, 2-way ANOVA test. $ P-value ≤ 0.05, $$ P-value ≤ 0.01, $$$$ P-value ≤ 0.0001 for drug-treated cells *vs* vehicle-treated (DMSO) cells, and *P-value ≤ 0.05, **P-value ≤ 0.01, ***P-value ≤ 0.001, ****P-value ≤ 0.0001 for drug combination-treated cells *vs* single agents.

Then, we evaluated whether a combinatorial approach by simultaneously inhibiting both NOTCH and MET signaling could potentiate the effects of single treatments on RMS cells. To this end, RD and RH30 cells were treated with PF-0308 and ARQ as single agents or in combination. As shown in **Figure 4A** and **Figure S6**, the combination significantly slowed down cell proliferation 72h post-treatment in all the RMS cell lines compared to each treatment alone, with more marked effects in FP-RMS cells.

In particular, the co-treatment resulted in a complete blockade of cell proliferation in RH30 cells (18.3 ± 0.5% and 17.2 ± 0.7% confluence at t0 and t72, respectively) (**Figure 4A**). In RD cells the drug combination resulted in ~62.0 ± 1.8% cell growth reduction compared to vehicle-treated cells (**Figure 4A**). Consistent with the reduction in cell confluence obtained with PF-0308 and ARQ as single agents, both inhibitors were able to decrease cell survival (percentage of Calcein-stained live cells) concomitantly increasing cell death (percentage of PI-stained dead cells) 72h post-treatment compared to cells treated with vehicle (**Figures 4B, C**). The drug combination further decreased cell survival and increased cell death in RH30 and RD cells *vs* single treatments (**Figures 4B, C**).

A very considerable decrease of cell growth in RH4 cells (83 ± 0.7% confluence) and a marked cell growth reduction reaching ~60 ± 4.3% in JR1 cells was also detected 72h post-treatment (**Figure S6**).

**Figure 5:**
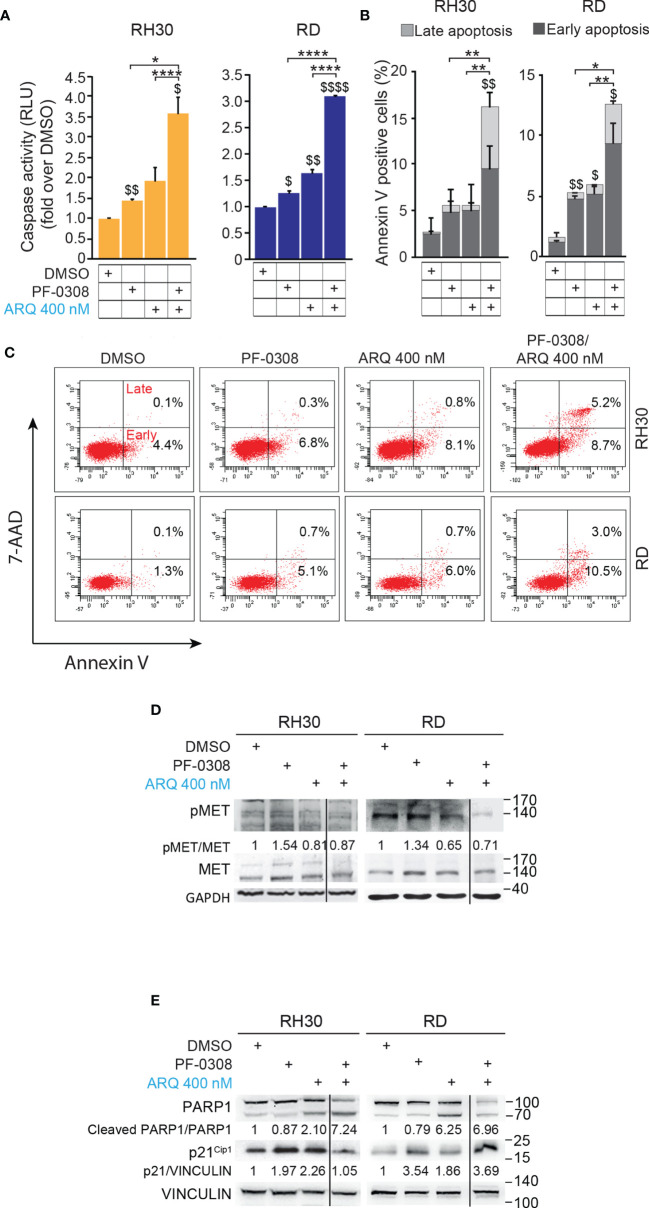
NOTCH and MET signaling co-inhibition induces caspase-dependent apoptosis. **(A)** Histograms depict Caspase-3/7 activity in RH30 and RD cells treated for 24h with either PF-03084014 (20 μM and 10 μM, respectively) or ARQ197 (400 nM) or with the drug combination. Graphs represent the mean of three independent experiments ± SD, 2-way ANOVA test. $ P-value ≤ 0.05, $$ P-value ≤ 0.01, $$$$ P-value ≤ 0.0001 for drug-treated cells *vs* vehicle-treated (DMSO) cells, and *P-value ≤ 0.05, ****P-value ≤ 0.001 for drug combination-treated cells *vs* single agents. **(B)** Graphs represent the mean of three independent experiments ± SD of Annexin V/7-AAD staining of RD and RH30 cells treated as in **(A)**. $ P-value ≤ 0.05, $$ P-value ≤ 0.01 drug-treated cells *vs* vehicle-treated (DMSO) cells, and *P-value ≤ 0.05, **P-value ≤ 0.01 for drug combination-treated cells *vs* single agents, 2-way ANOVA test. **(C)** Representative cytofluorimetric plots showing Annexin V/7-AAD staining of RH30 and RD cells treated as in **(A)**. Dot plots depict the percentage of Annexin-V/7-AAD single- and double-positive cells. **(D)** Representative radiographs of RH30 and RD cells treated with either PF-03084014 (20 μM for 48h and 10 μM for 72h, respectively) or ARQ197 (400 nM) or with the drug combination. Radiographs show total and phosphorylated (pMET^Y1234/1235^) MET protein levels. Migration of molecular weight markers is indicated on the right (kDa). GAPDH was used as loading control. **(E)** Representative radiographs of RH30 and RD cells treated with either PF-03084014 (20 μM for 48h and 10 μM for 72h, respectively) or ARQ197 (400 nM) or with the drug combination. Radiographs show PARP1 and p21^Cip1^ protein levels were also showed. Migration of molecular weight markers is indicated on the right (kDa). VINCULIN was used as loading control.

Then, we, evaluated whether the reduction in cell proliferation and the increase in PI-positive cells could be due to apoptotic cell death by measuring caspase 3/7 (CASP3/7) cell activity and Annexin V cell positivity, two markers of apoptosis. As shown in **Figure 5A** and **Figure S7A**, the drug combination significantly and markedly increased CASP3/7 activation in all the cell lines compared to each single agents, which individually were effective compared to vehicle. In line with these findings, the drug combination considerably increased Annexin V cell positivity compared to single treatments in all cell lines (**Figures 5B, C** and **Figures S7B, C**).

Interestingly, the percentage of Annexin V-positive cells was significantly enhanced by both inhibitors as single agents in RH4 and RD cells compared to vehicle. On the other hand, Annexin V-positive cells were increased only by ARQ in JR1 cells and did not reach the significance after both individual treatments in RH30 cells (**Figures 5B, C** and **Figures S7B, C**).

At the molecular level, combined treatment counteracted the increase of phosphorylated pMET due to PF-0308 and maintained pMET levels lower than those of the vehicle in all four cell lines (**Figure 5D** and **Figure S7D**). These results indicated that the combination treatment was able to counteract MET activation due to NOTCH inhibition.

**Figure 6:**
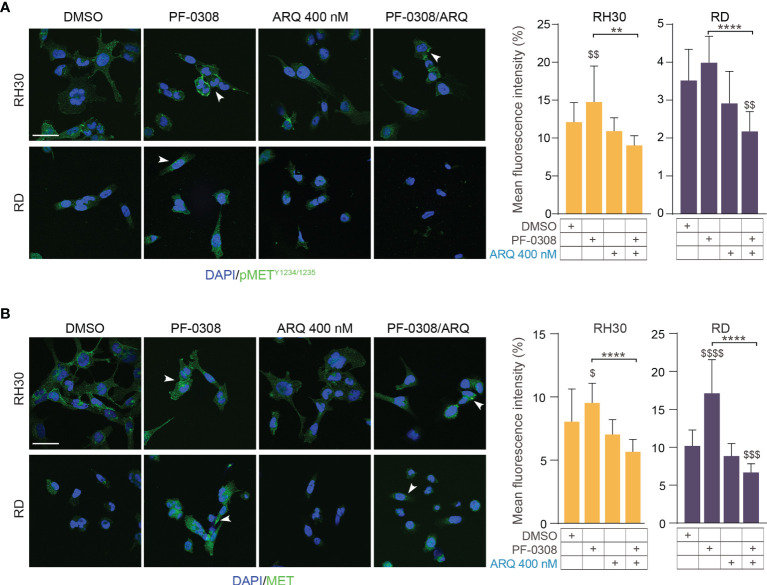
NOTCH and MET signaling co-inhibition affects MET expression and localization.0 **(A)** (left) Representative immunofluorescence images of pMET^Y1234/1235^ (green) in RH30 and RD cells treated for 8h with either PF-03084014 (20 μM and 10 μM, respectively), or ARQ197 400 nM, or with the drug combination. DAPI (blue) was used for nuclear staining. (right) Histograms depicting % of pMET mean fluorescence intensity of RH30 and RD cells treated for 8h with either PF-03084014 (20 μM and 10 μM, respectively), or ARQ197 400 nM, or with the drug combination ± SD, 2-way ANOVA test. $$ P-value ≤ 0.01 drug-treated cells *vs* vehicle-treated (DMSO) cells, and **P-value ≤ 0.01, ****P-value ≤ 0.0001 for drug combination-treated cells *vs* single agents. **(B)** (left) Representative immunofluorescence images of total MET (green) in RH30 and RD cells treated as in **(A)** for 24h. DAPI (blue) was used for nuclear staining. 60X magnification; scale bar 50 μM. (right) Histograms depicting % of total MET mean fluorescence intensity of RH30 and RD cells treated for as in **(A)** ± SD, 2-way ANOVA test. $ P-value ≤ 0.05, $$$ P-value ≤ 0.001, $$$$ P-value ≤ 0.0001 drug-treated cells *vs* vehicle-treated (DMSO) cells, and ****P-value ≤ 0.001 for drug combination-treated cells *vs* single agents.

Moreover, PF-0308 did not affect cleaved PARP1 levels while ARQ treatment strongly promoted PARP1 cleavage compared to vehicle, which is more marked in RH30 and RD cell lines (**Figure 5E** and **Figure S7E**). The drug combination dramatically enhanced this effect compared to single ARQ treatment in RH30, JR1 and RH4 cells (**Figure 5E** and **Figure S7E**). Conversely, increased PARP1 cleavage due to drug combination in RD cells was similar to single ARQ treatment (**Figure 5E**). This suggests that, in RD cells, the levels of PARP1 cleavage by the combination relies mostly on ARQ in the used experimental conditions. Moreover, the levels of p21^Cip1^ were not significantly affected by the two combinations *vs* single agents in all cell lines (**Figure 5E** and **Figure S7E**). The effects of treatments on the expression and localization of pMET/MET were then investigated in RH30 and RD cells by immunofluorescence (IF). **Figure 6A** shows that the staining for MET phosphorylation in vehicle-treated cells was mainly localized in the cytoplasm in both cell lines and it was more marked in RH30 cells than in RD cells.

After PF-0308 treatment, pMET staining was localized more on the cell membrane especially in RH30 cells that also showed an increase in the expression levels, as indicated by the significant enhancement of the intensity of fluorescence *vs* vehicle-treated cells (**Figure 6A**). Conversely, ARQ reduced the intensity of pMET staining in both cell lines even if values did not reach the significance (**Figure 6A**). The drug combination counteracted the increase in pMET due to PF-0308 in both cell lines compared to NOTCHi alone and impaired its localization on the cell membrane (**Figure 6A**). In line with the immunoblot assays results, PF-0308 individually increased total MET staining and the combination was able to impair the PF-0308-inducer effect on MET expression and localization in both cell lines (**Figure 6B**).

Overall, these results suggest that co-inhibition of NOTCH and MET signaling amplifies the anti-proliferative effects of single pathways inhibition in RMS cells partly through the induction of apoptosis.

### Combined Inhibition of NOTCH and MET Signaling Impairs the Tumorigenic Properties of RMS Cells

**Figure 7:**
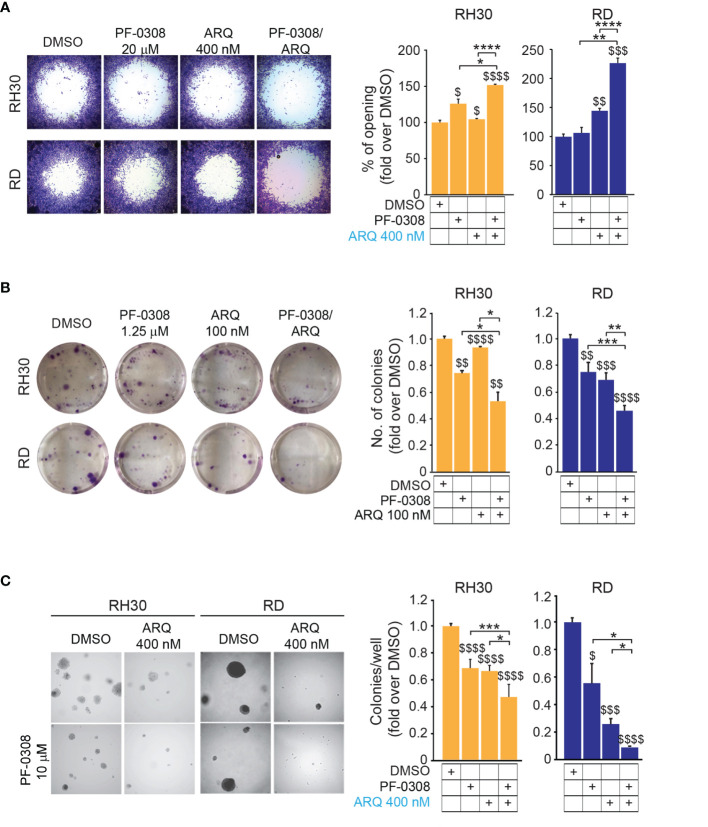
NOTCH and MET signaling co-inhibition reduces migration and tumorigenicity *in vitro*. **(A)** (left) Representative images of a migration assay of RH30 and RD cells treated for 24h with either PF-03084014 (20 μM and 10 μM, respectively) or ARQ197 400 nM or with the drug combination. (right) Histograms depict the percentage of the opening area in RH30 and RD cells. Graphs represent the mean of three independent experiments ± SD, 2-way ANOVA test. $ P-value ≤ 0.05, $$ P-value ≤ 0.01, $$$ P-value ≤ 0.001, $$$$ P-value ≤ 0.0001 for drug-treated cells *vs* vehicle-treated (DMSO) cells, and *P-value ≤ 0.05, **P-value ≤ 0.01, ****P-value ≤ 0.0001 for drug combination-treated *vs* single agent-treated cells. **(B)** (left) Representative images of a colony formation assay of RH30 and RD cells treated with either PF-03084014 1.25 μM or ARQ197 100 nM or with the drug combination. (right) Histogram of quantitation values of colony forming units. Graphs represent the mean of three independent experiments ± SD, 2-way ANOVA test. $$ P-value ≤ 0.01, $$$ P-value ≤ 0.001, $$$$ P-value ≤ 0.0001 for drug-treated cells *vs* vehicle-treated (DMSO) cells, and *P-value ≤ 0.05, **P-value ≤ 0.01, ***P-value ≤ 0.001 for drug combination-treated cells *vs* single agents. **(C)** (left) Representative images of a soft agar colony formation assay of RH30 and RD cells treated with either PF-03084014 (20 μM and 10 μM, respectively) or ARQ197 400 nM or with the drug combination. (right) Histograms of colony numbers/wells quantitation. Graphs represent the mean of three independent experiments ± SD, 2-way ANOVA test. $ P-value ≤ 0.05, $$$ P-value ≤ 0.001, $$$$ P-value ≤ 0.0001 for drug-treated cells *vs* vehicle-treated (DMSO) cells, and *P-value ≤ 0.05, **P-value ≤ 0.01, ***P-value ≤ 0.001 for drug combination-treated cells *vs* single agents.

Since both NOTCH and MET pathways regulate RMS cell migration (16, 40), we evaluated the migratory phenotype of the four cell lines after pharmacologic co-inhibition of the two pathways. We used a short time point, i.e., 24h, and a low serum condition (see *Materials and Methods*) to avoid the confounding effects of the drugs on cell proliferation. The NOTCH inhibitor PF-0308 as single agent was able to significantly reduce the migration of RH30 and JR1 cells relative to the vehicle, as indicated by the increase in the cell-free area, while this effect was not significant neither in RD nor in RH4 cells, or rather in the latter the migration even seemed to be slightly increased (**Figure 7A** and **Figure S8A**). MET inhibition with ARQ enhanced the cell-free area in three cell lines with the exception of RH4 cells, while the combination significantly hampered the migratory phenotype of the four cell lines compared to single agents (**Figure 7A** and **Figure S8A**).

We then tested the capacity of RMS cells to grow into single cell-derived colonies through a clonogenic assay. Using drug concentrations that allowed the formation of a measurable number of cell colonies in single agent-treated cells (i.e., PF-0308 1.25 µM and ARQ 100 nM) we observed that each drug was able to lower the number of colonies *vs* vehicle, with the condition of drug combination that further decreased colony formation (**Figure 7B** and **Figure S8B**). The capability of tumor cells to grow in an anchorage-independent manner, as a stringent feature of malignant transformation, was also determined by measuring the number of colonies grown in soft-agar. Single treatments significantly affected the capacity of RMS cells to form colonies relative to vehicle-treated cells, and this effect was further amplified by PF-0308 + ARQ in the four cell lines (**Figure 7C** and **Figure S8C**).

**Figure 8:**
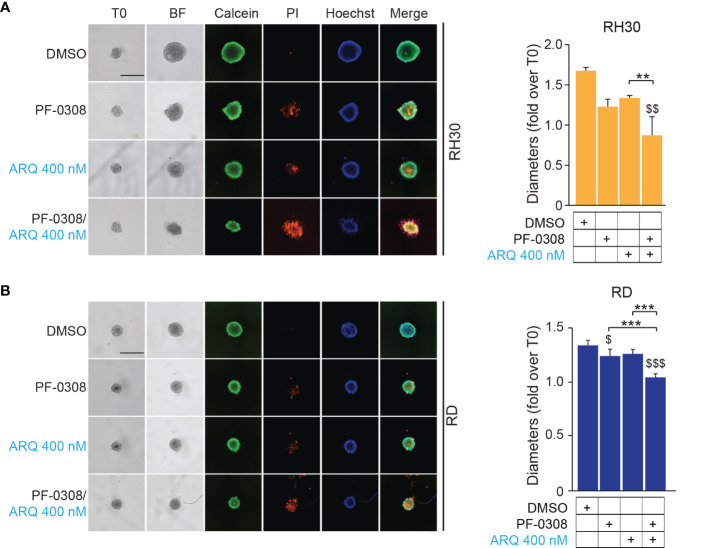
NOTCH and MET signaling co-inhibition induces growth arrest and cell death in RMS tumor spheroids. **(A)** (left) Representative images of RH30 tumor spheroids treated for 72h with PF-03084014 (20 μM) and ARQ197 (400 nM), alone or in combination. Scale bars = 500 μm. (right) Histogram of spheroids’ diameters quantification in RH30 cells. Graphs represent the mean of three independent experiments ± SD, 2-way ANOVA test. $$ P-value ≤ 0.01 for drug-treated cells *vs* vehicle-treated (DMSO) cells and **P-value ≤ 0.01 for drug combination-treated cells *vs* single agents. **(B)** (left) Representative images of RD tumor spheroids treated for 72h with PF-03084014 (10 μM) and ARQ197 (400 nM), alone or in combination. Scale bars = 500 μm. (right) Histogram of spheroids’ diameters quantification in RD cells. Graphs represent the mean of three independent experiments ± SD, 2-way ANOVA test. $ P-value ≤ 0.05, $$$ P-value ≤ 0.001 for drug-treated cells *vs* vehicle-treated (DMSO) cells, and ***P-value ≤ 0.001 for drug combination-treated cells *vs* single agents.

Then, we cultured RMS cells as spheroids (see *Materials and Methods*) to allow the formation of 3D structures more reminiscent of the *in vivo* growth, and treated them with drugs. PF-0308 as single agent significantly reduced spheroids growth *vs* vehicle-treated cells in RD, RH4 and JR1 cells, as shown by the decrease in spheroids’ diameter calculated on Calcein-stained live cells, while ARQ appeared ineffective (**Figures 8A, B** and **Figures S9A, B**).

Although the combination significantly inhibited 3D cell growth in the four cell lines compared to the vehicle, the diameter of spheroids was significantly reduced compared to both single treatments in RD, RH4 and JR1 cells while its effects were significant only when compared to ARQ in RH30 cells (**Figures 8A, B** and **Figures S9A, B**). Of note, both drugs induced the appearance of a PI-positive dead cell population, suggesting a pro-death effect in RMS cell lines growing in 3D, which was strongly increased by the combinations (**Figures 8A, B** and **Figures S9A, B**).

Altogether, our findings demonstrate that combining NOTCH with MET inhibition severely hampers the migratory and *in vitro* tumorigenic properties of RMS cells compared to single treatments.

## Discussion

NOTCH and MET signaling have been demonstrated pro-oncogenic in RMS and their genetic or pharmacologic inhibition prevents tumor cell growth and survival *in vitro* and *in vivo* (16–18, 40–42, 45, 47, 48). It has been reported that the MET oncogene can be repressed by NOTCH signaling in breast cancer (31). Moreover, NOTCH blockade using γ-secretase inhibitors have been shown unable to induce sustained anti-tumor effects in clinical studies [reviewed in (20)] suggesting mechanisms of resistance restraining their efficacy (53–57). Based on these evidences, in the present work we investigated whether MET is modulated in response to NOTCH inhibition in RMS with the aim to evaluate the effects of a combined NOTCH-MET inhibitory approach.

Our results show that *in vitro* blockade of the NOTCH pathway using the γ-secretase inhibitor PF-03084014 (PF-0308), investigated in clinical trials on other tumor types, resulted in the up-regulation of total and phosphorylated MET protein levels in both the FP-RMS RH30 and RH4 and the FN-RMS RD and JR1 cell lines.

Interestingly, MET mRNA levels were unmodified by the γ-secretase inhibitor in RH30 and slightly decreased in RH4 cells, suggesting that non-transcriptional mechanisms could be involved in the observed phenomenon in the FP-RMS subtype. Conversely, in the FN-RMS RD and JR1 cells MET transcripts increased after pharmacologic NOTCH signaling suppression suggesting transcriptional mechanisms concur to the enhancement of MET protein levels in this RMS subtype.

Notably, another different γ-secretase inhibitor, i.e., GSI-XII, also up-regulated MET levels when tested on RH30 and RD cells suggesting that this phenomenon is a general response to NOTCH signaling inhibition in RMS cells. Moreover, MET protein overexpression was also observed by individually silencing NOTCH1 and NOTCH3 receptors in all four cell lines indicating the effect was specifically due to NOTCH signaling inactivation. Of note, while MET transcript levels remained unaltered in FP-RMS cell lines after silencing of each NOTCH paralogue, they significantly increased in both FN-RMS cell lines suggesting that the blockade of both receptors, as after γ-secretase inhibition, is needed for a transcriptional effect.

Since in addition to MET amplification or mutation, MET pathway overexpression and hyper-activation has been correlated to the emergence of drug resistance after targeted and anti-kinase therapy [reviewed by (58, 59)]. So, we investigated the co-inhibition of NOTCH and MET pathways in our tumor cell context to evaluate potential synergies on malignant cell features. To this end, we screened five MET inhibitors already in clinical trials or previously used on RMS cells (45–47), and we decided to test ARQ197 (Tivantinib) due to the IC50 nanomolar range.

As already reported for other types of cancer cells (60), ARQ treatment showed a specific effect on MET in our cell lines since it lowered MET phosphorylation, suggesting down-regulation of MET activity. We show here that both the γ-secretase and MET inhibitors as single agents had anti-proliferative effects associated to p21^Cip1^ increase in both RMS cell subtypes.

However, although significant activation of caspase 3/7 was induced by NOTCHi in all four cell lines, it was able to significantly increase the percentage of Annexin V positive cells only in RD and RH4 cell lines. Similarly, a cell line-specific response was seen for ARQ, which activated caspase 3/7 and enhanced Annexin V positivity in three cell lines with the exception of RH30 cells. Furthermore, only METi promoted PARP1 cleavage in all the tested cell lines, even if the increase in cleavage is mild in RH4 cells. These results suggest that NOTCHi induces PARP1-independent while METi PARP1-dependent apoptosis in RMS cells. In RH30 and JR1 cells, where neither Annexin V positivity nor PARP-1 cleavage increase was seen in response to NOTCHi, cell death could be due to a transient cell activation of caspase 3/7 (61) or, alternatively, to an heterogeneous caspase 3/7 activation in some cell subpopulations (62). Further, these data also demonstrate that the response to pan-NOTCHi seems to be independent of the RMS subtype.

All these effects were significantly enhanced combining the two inhibitors. Indeed, in all the RMS cell lines treated with the combination, the levels of MET and its phosphorylated form were similar or even decreased compared to those in vehicle-treated control cells demonstrating that ARQ in the combination counteracted the PF-0308-induced increase of MET protein levels. In addition, the RMS cell lines proliferated significantly less than control cells under drug combination, with the stronger effect on FP-RMS cells. Furthermore, NOTCH-MET co-inhibition was capable of triggeringsustained apoptosis in RMS cells as testified by the marked enhancement of the activity of caspase 3/7 and the concomitant augmentation of Annexin V positivity and PARP1 cleavage *vs* each single drug. These findings suggest that this combinatorial targeting triggers apoptotic cell death, which is dependent by PARP1 in RMS cells.

Moreover, while single treatments mildly affected the capacity of RMS cells to migrate, form colonies, and grow in an anchorage-independent manner, the PF-0308 and ARQ combination had a remarkable inhibitory effect on these tumorigenic properties. NOTCH-MET co-inhibition also strongly affected the survival of RMS cells grown as spheroids increasing cell death as compared to individual drug treatments. The drug combination showed a different impact on the proliferation ability of the two RMS subtypes resulting in a larger inhibitory effect than the sum of the effects of each individual drug in FP-RMS but not in FN-RMS cells. Further, the marked activation of caspase 3/7 and the induction of Annexin V positivity in three cell lines were greater than that due to additive effects, with the exception of RH4 cells. However, further *in vitro* studies are needed to clarify whether the pharmacological co-inhibition of NOTCH and MET pathways have synergistic or additive effects or both on the different malignant features in our tumor context.

Recently, ARQ has shown partial results in clinical trials on patients with MET overexpression or amplification (63). Further, *in vitro* studies showed that ARQ affects cell proliferation in cells not expressing MET and does not inhibit MET phosphorylation in lung cancer cells with low basal levels of MET expression (64, 65) or in gastric carcinoma cells characterized by MET amplification and constitutive MET activation (66). In these cell contexts, its anti-tumor function has been correlated with the inhibition of molecular targets other than MET (64, 67). However, in RMS cell lines the reduction in both total and phosphorylated forms of MET seem to suggest that ARQ is capable of targeting the oncogene, as suggested by previous reports (60). In addition, in the high-risk RMS tumor cells used in the present work (68, 69) MET is basally overexpressed and activated and NOTCHi further increases its activation thus possibly making it a major target of ARQ inhibition.

Of note, the doses of the drugs used in the present work are in line with the literature. Indeed, the NOTCHi has been recently shown to inhibit cell renewal and proliferation of HCC cancer stem cells using doses up to 15 μM *in vitro* and to block tumor growth *in vivo* (70). Further, ARQ doses were similar to those that induced pMET down-regulation in several cancer cell lines and inhibit tumor growth *in vivo* (60). The inhibitors have been used for the first time in combination in the present work, so no data are available on the toxicity *in vivo* for this combination, which needs to be verified in preclinical models in future studies.

In conclusion, we unveil a novel mechanism of MET induction that on the one hand could potentially influence the anti-cancer effects of NOTCH signaling blockade in RMS cells and, on the other hand, could represent a novel vulnerability that can be targeted. In line with this, our results demonstrated that inhibiting both NOTCH and MET signaling overcomes the activation of MET following NOTCH signaling restriction and, even if they should be further evaluated in future preclinical studies, they deserve attention as they can have a translational impact.

## Data Availability Statement

The original contributions presented in the study are included in the article/**Supplementary Material**. Further inquiries can be directed to the corresponding author.

## Author Contributions

CP, MCa, and SPo performed and/or interpreted or supervised the experimental aspects. CP, SPo, MCa, CC, MP, MCo performed wet experiments and acquired result data. GP and MV helped with the 3D model. SPe performed confocal microscopy. JR, SG, and LM helped on the interpretation of NOTCH signaling inhibition. GMM, FM, CQ, SC, ADG, BDA, EdB, PM, RT, and MCe participated in the analysis of the data and provided reagents. RM, LM, and FL participated in the writing and critically revising the manuscript. RR designed and supervised the study, wrote and revised the final version of the manuscript. All authors read and approved the final version of the manuscript.

## Funding

This research was funded by Associazione Italiana per la Ricerca sul Cancro (AIRC) #15312 to RR and #24696 to FM; Italian Ministry of Health (Ricerca Corrente) to BDA, CQ, and RR; AIRC 5xmille #9962 to FL; Italian Ministry of Health (Fondi 5xmille 2021-2022) to RR; Alleanza Contro il Cancro (ACC) Italian Network-Working Group Sarcomas to BDA, RM, and RR; Fondi Ateneo 2019 to FM; MIUR-Italy: Grant to Department of Science, Roma Tre University (Dipartimento di Eccellenza, ARTICOLO 1, COMMI 314—337 LEGGE 232/2016) to MCe and PM.

## Conflict of Interest

The authors declare that the research was conducted in the absence of any commercial or financial relationships that could be construed as a potential conflict of interest.

## Publisher’s Note

All claims expressed in this article are solely those of the authors and do not necessarily represent those of their affiliated organizations, or those of the publisher, the editors and the reviewers. Any product that may be evaluated in this article, or claim that may be made by its manufacturer, is not guaranteed or endorsed by the publisher.
