# Supplementary material for: MET Inhibition Sensitizes Rhabdomyosarcoma Cells to NOTCH Signaling Suppression
Source: Front Oncol. 2022 Apr 27;12:835642. doi: 10.3389/fonc.2022.835642 (PMC9092259; doi:10.3389/fonc.2022.835642)
Supplement: Supplementary file 1 [file DataSheet_1.docx]

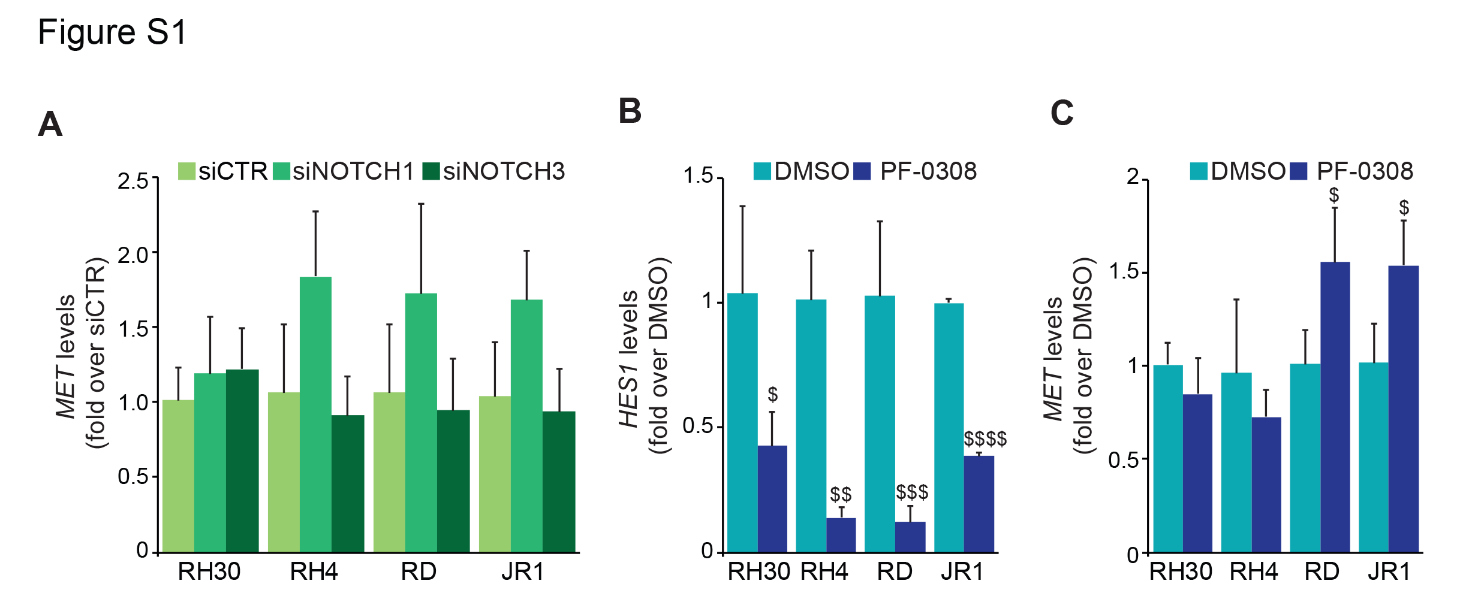


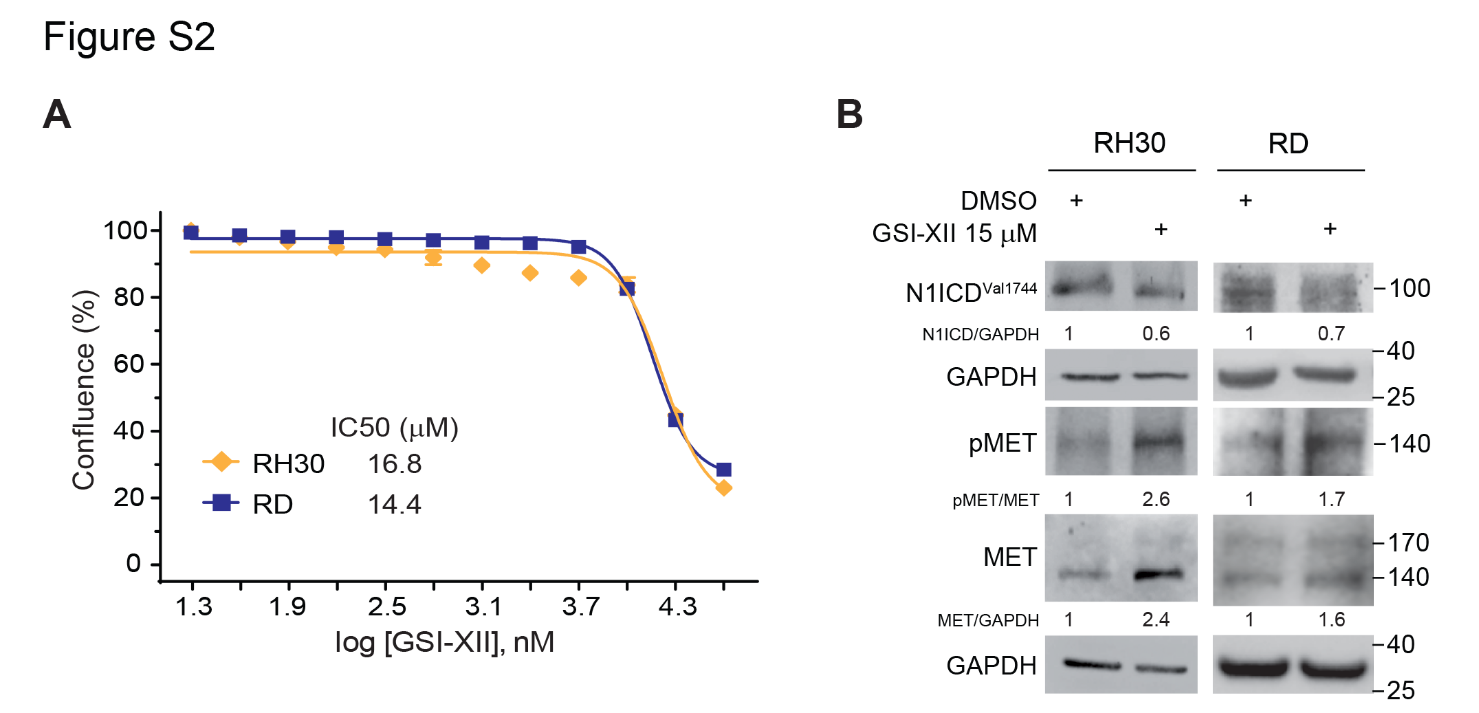
**Supplementary Figure 1. NOTCH signaling inhibition drives MET up-regulation in RMS cells.** **(A)** Effect of NOTCH1 and NOTCH3 silencing on *MET* mRNA levels in RH30, RH4, RD and JR1 cells. Graph represents the mean of three independent experiments ± SD. qRT-PCR of **(B)** *HES1* and **(C)** *MET* in RH30 and RH4 cells treated with 20 μM for 48h, and in RD and JR1 cells treated with 10 μM for 72h of PF-03084014. Graph represents the mean of three independent experiments ± SD, Student two-tailed T-Test. $ P-value ≤ 0.05, $$ P-value ≤ 0.01, $$$ P-value ≤ 0.001, $$$$ P-value ≤ 0.0001 treated cells *vs* DMSO.

**Supplementary Figure 2. GSI-XII NOTCH inhibitor induces MET activation/up-regulation in RMS cells. (A)** Dose-response curves of RH30 and RD cells treated with NOTCH inhibitor GSI-XII. Graph represents the mean of three independent experiment ± SEM. **(B)** Representative (n=3 independent experiments) Western Blot depicting the effect of 15 μM for 48h (RH30) and 72h (RD) of GSI-XII treatment on MET activation (pMET ^Y1234/1235^) and total levels (MET) in RH30 and RD cells, respectively. Migration of molecular weight markers is indicated on the right (kDa). N1ICD^Val1744^ protein level was used as treatment control. GAPDH levels were the loading control.

**
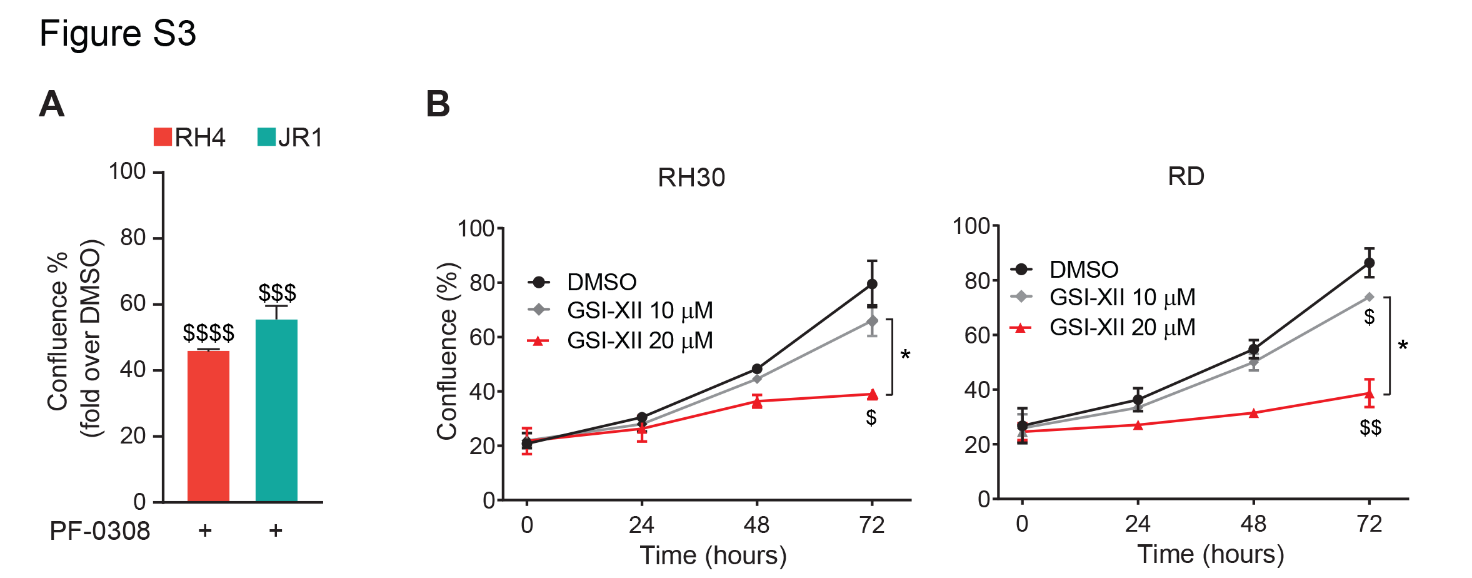
**

**Supplementary Figure 3. Pharmacologic inhibition of NOTCH signaling hampers RMS cells growth.** **(A)** Histogram depicting the effect of PF-03084014 20 μM and 10 μM for 72h on RH4 and JR1 cells, respectively. Graph represents the mean of three independent experiments ± SD, Student two-tailed T-Test. $$$ P-value ≤ 0.001, $$$$ P-value ≤ 0.0001 treated cells *vs* DMSO. **(B)** Growth-curves of RH30 and RD treated with 10 μM and 20 μM of GSI-XII. Graph represents the mean of three independent experiments ± SD, 2-way ANOVA test. $ P-value ≤ 0.05, $$ P-value ≤ 0.01 for GSI-XII *vs* DMSO and *P-value ≤ 0.05 for 20 μM GSI-XII *vs* 10 μM GSI-XII.


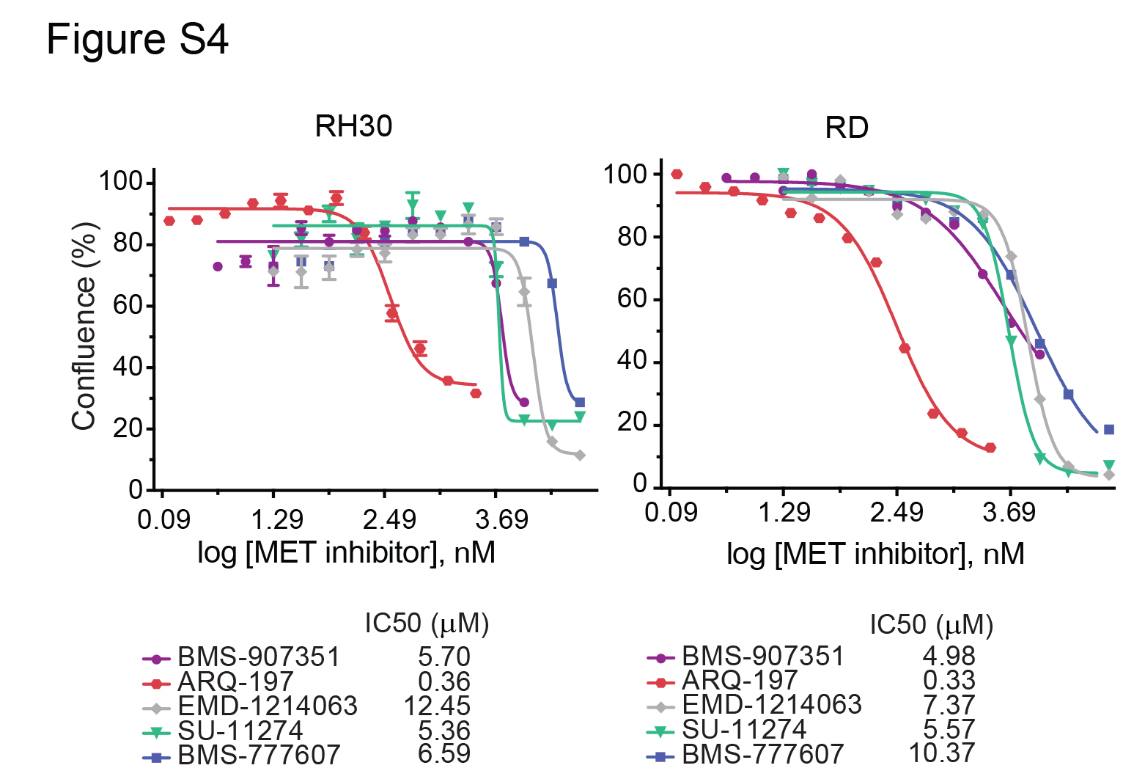


**Supplementary Figure 4. Effect of MET inhibitors on RH30 and RD RMS cell lines.** **(A)** Dose-response curves of RH30 and RD cells treated with a panel of MET inhibitors. Graph represents the mean of three independent experiment ± SEM.


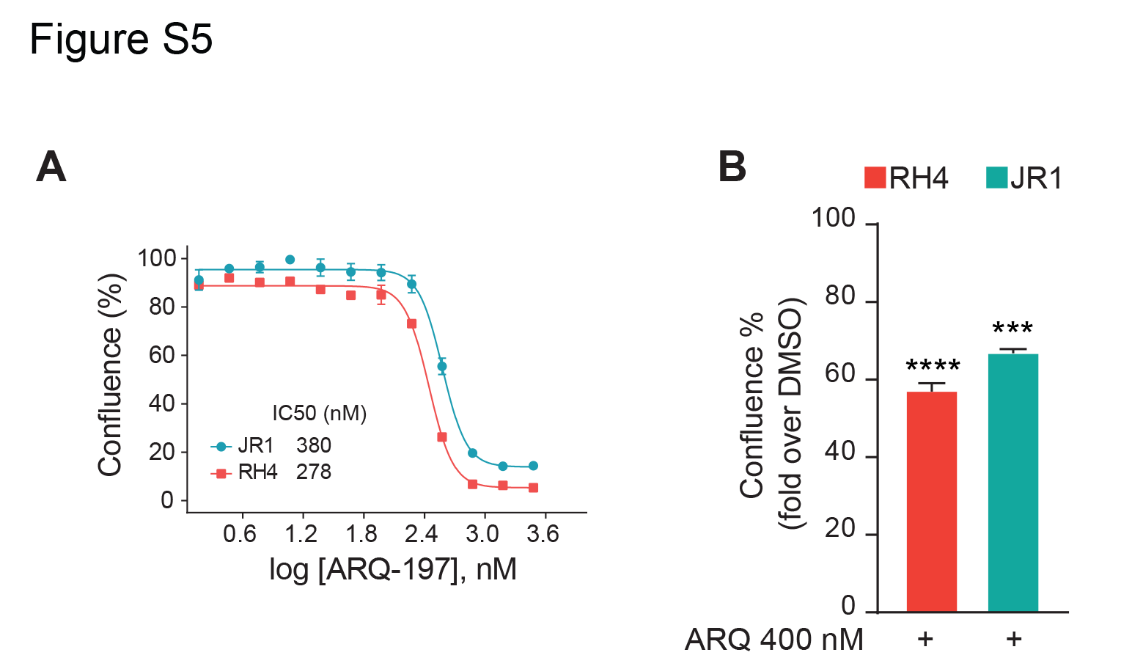


**Supplementary Figure 5. Effect of MET inhibitor, ARQ-197, on the growth of RH4 and JR1 RMS cell lines.** **(A)** Dose-response curves of RH30 and RD cells treated with ARQ-197. Graph represents the mean of three independent experiment ± SEM. **(B)** Histogram depicting the effect of 72h ARQ-197 400 nM treatment on RH4 and JR1 cells. Graph represents the mean of three independent experiments ± SD, Student two-tailed T-Test. *** P-value ≤ 0.001, **** P-value ≤ 0.0001 treated cells *vs* DMSO.


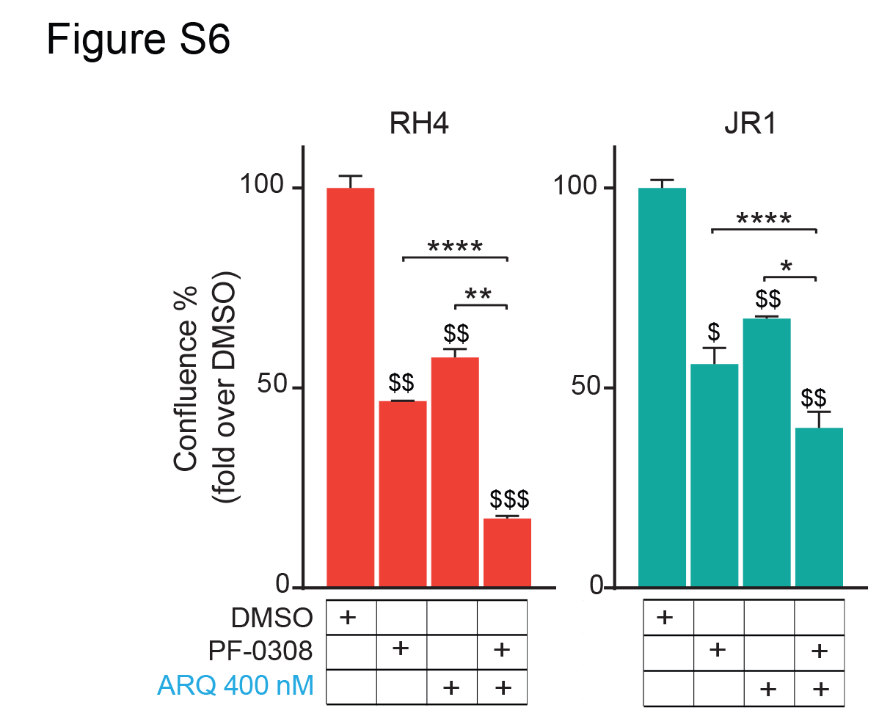


**Supplementary Figure 6. NOTCH and MET signaling co-inhibition hampers RMS cells growth.** Histograms depicting the effect of 20 µM (RH4) or 10 µM (JR1) of PF-03084014, or 400 nM of ARQ197 or with the drug combination for 72h. Graphs represent the mean of three independent experiments ± SD, 2-way ANOVA test. $ P-value ≤ 0.05, $$ P-value ≤ 0.01, $$$ P-value ≤ 0.001 treated cells *vs* DMSO and * P-value ≤ 0.05, ** P-value ≤ 0.01, **** P-value ≤ 0.0001 for drug combination-treated cells *vs* single agents.

**
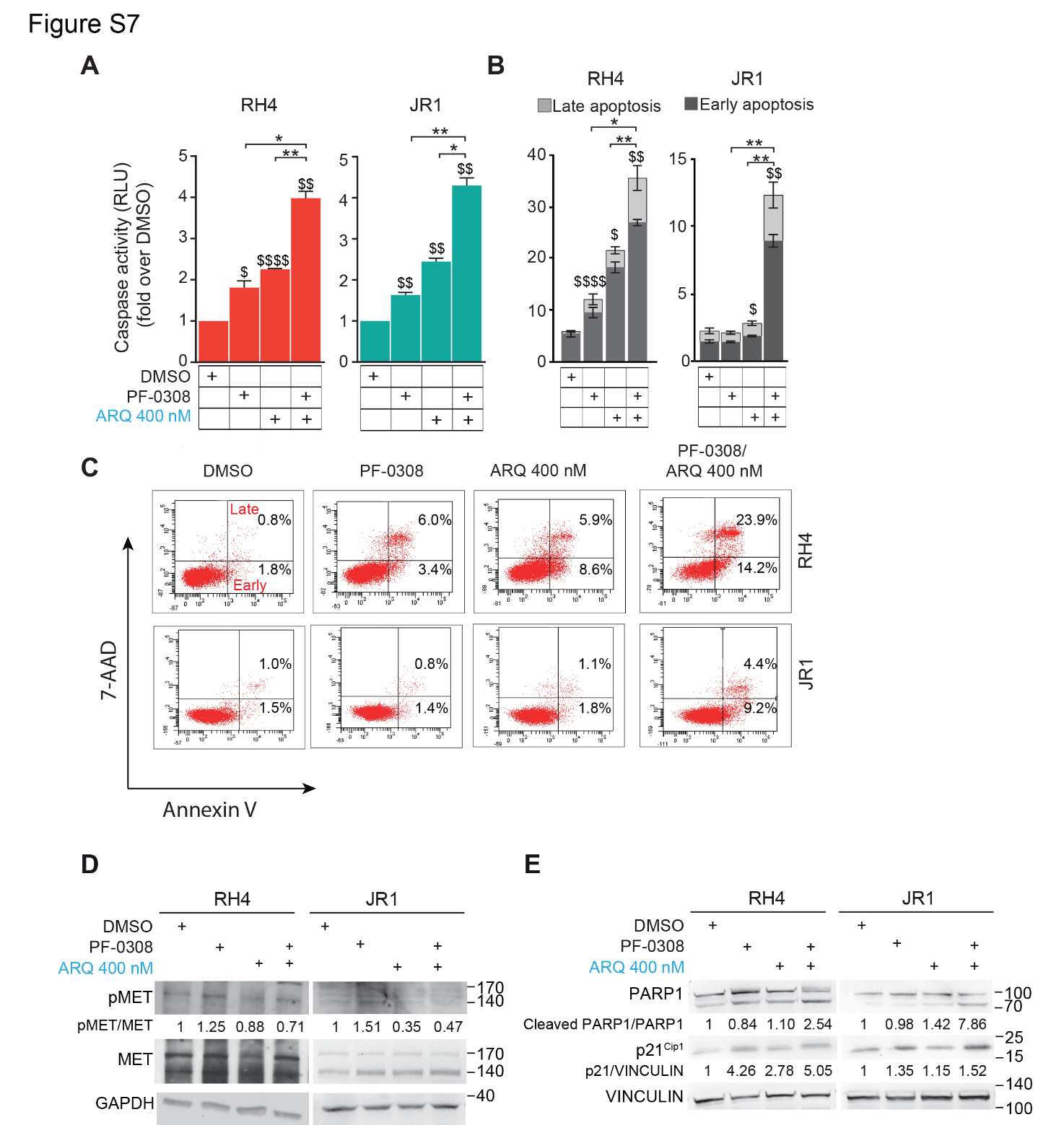
**

**Supplementary Figure 7. NOTCH and MET signaling co-inhibition induces caspase-dependent apoptosis. (A)** Histograms depict Caspase-3/7 activity in RH4 and JR1 cells treated for 24h with either PF-03084014 (20 μM and 10 μM, respectively) or ARQ197 (400 nM) or with the drug combination. Graphs represent the mean of three independent experiments ± SD, 2-way ANOVA test. $ P-value ≤ 0.05, $$ P-value ≤ 0.01, $$$$ P-value ≤ 0.0001 for drug-treated cells *vs* vehicle-treated (DMSO) cells, and * P-value ≤ 0.05, ** P-value ≤ 0.01 for drug combination-treated cells *vs* single agents. **(B)** Graphs represent the mean of three independent experiments ± SD of Annexin V/7-AAD staining of RH4 and JR1 cells treated as in **(A)**. $ P-value ≤ 0.05, $$ P-value ≤ 0.01, $$$$ P-value ≤ 0.0001 drug-treated cells *vs* vehicle-treated (DMSO) cells, and * P-value ≤ 0.05, ** P-value ≤ 0.01 for drug combination-treated cells *vs* single agents, 2-way ANOVA test. **(C)** Representative cytofluorimetric plots showing Annexin V/7-AAD staining of RH4 and JR1 cells treated as in **(A)**. Dot plots depict the percentage of Annexin-V/7-AAD single- and double-positive cells. (**D)** Representative radiographs of RH4 and JR1 cells treated with either PF-03084014 (20 μM for 48h and 10 μM for 72h, respectively) or ARQ197 (400 nM) or with the drug combination. Radiographs show total and phosphorylated (pMET^Y1234/1235^) MET protein levels. Migration of molecular weight markers is indicated on the right (kDa). GAPDH was used as loading controls. **(E)** Representative radiographs of RH4 and JR1 cells treated as in **(D)**. PARP1 and p21^Cip1^ protein levels were showed. Migration of molecular weight markers is indicated on the right (kDa). VINCULIN was used as loading controls.


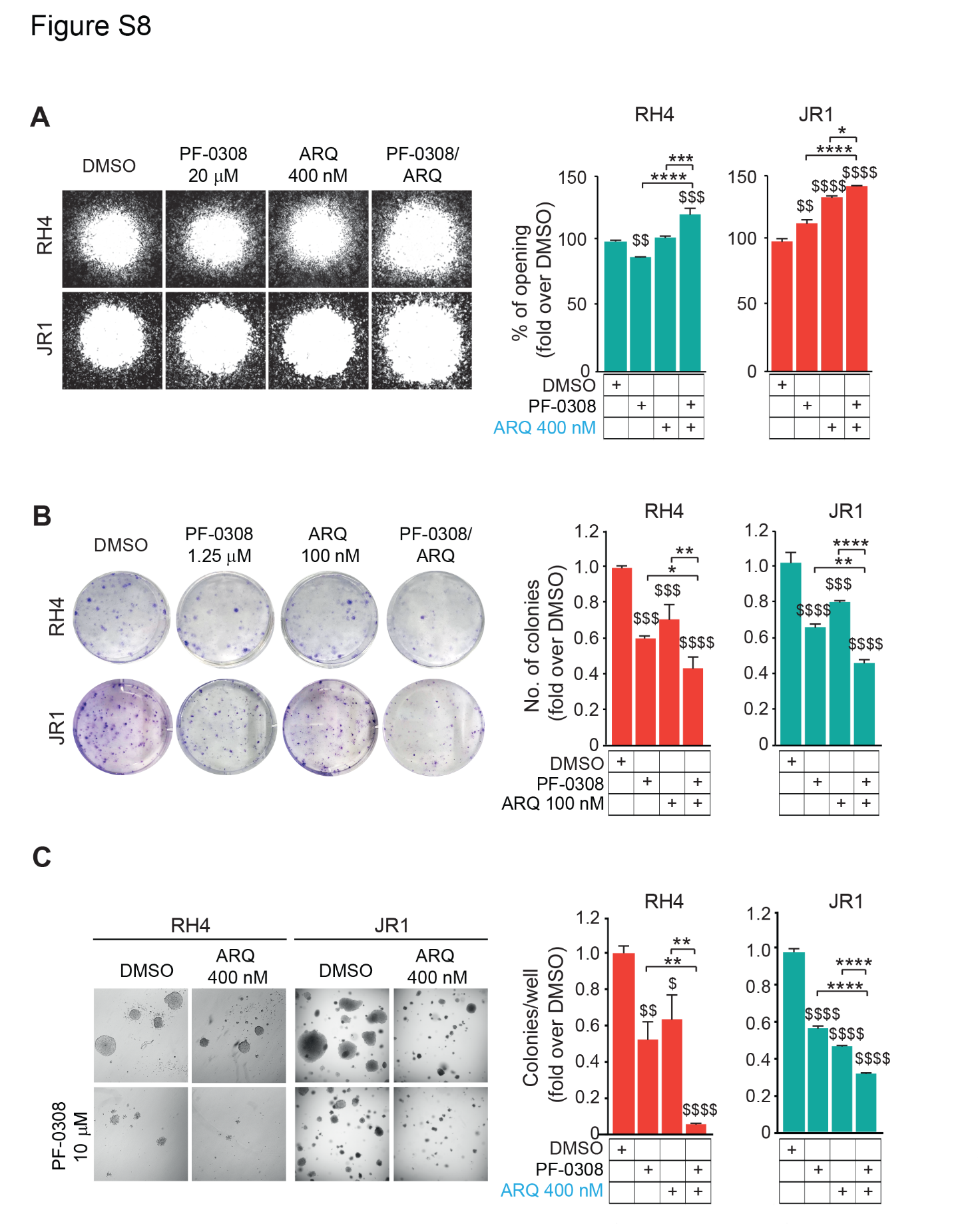


**Supplementary Figure 8. NOTCH and MET signaling co-inhibition reduces migration and tumorigenicity *in vitro*. (A) (left)** Representative images of a migration assay of RH4 and JR1 cells treated for 24h with either PF-03084014 (20 μM and 10 μM, respectively) or ARQ197 400 nM or with the drug combination. **(right)** Histograms depict the percentage of the opening area in RH4 and JR1 cells. Graphs represent the mean of three independent experiments ± SD, 2-way ANOVA test. $$ P-value ≤ 0.01, $$$ P-value ≤ 0.001, $$$$ P-value ≤ 0.0001 for drug-treated cells *vs* vehicle-treated (DMSO) cells, and * P-value ≤ 0.05, *** P-value ≤ 0.001, **** P-value ≤ 0.0001 for drug combination-treated *vs* single agent-treated cells. **(B)** **(left)** Representative images of a colony formation assay of RH4 and JR1 cells treated with either PF-03084014 1.25 μM or ARQ197 100 nM or with the drug combination. **(right)** Histogram of quantitation values of colony forming units. Graphs represent the mean of three independent experiments ± SD, 2-way ANOVA test. $$$ P-value ≤ 0.001, $$$$ P-value ≤ 0.0001 for drug-treated cells *vs* vehicle-treated (DMSO) cells, and * P-value ≤ 0.05, ** P-value ≤ 0.01, **** P-value ≤ 0.0001 for drug combination-treated cells *vs* single agents. **(C)** **(left)** Representative images of a soft agar colony formation assay of RH4 and JR1 cells treated with either PF-03084014 (20 μM and 10 μM, respectively) or ARQ197 400 nM or with the drug combination. **(right)** Histograms of colony numbers/wells quantitation. Graphs represent the mean of three independent experiments ± SD, 2-way ANOVA test. $ P-value ≤ 0.05, $$ P-value ≤ 0.01, $$$$ P-value ≤ 0.0001 for drug-treated cells *vs* vehicle-treated (DMSO) cells, and ** P-value ≤ 0.01, **
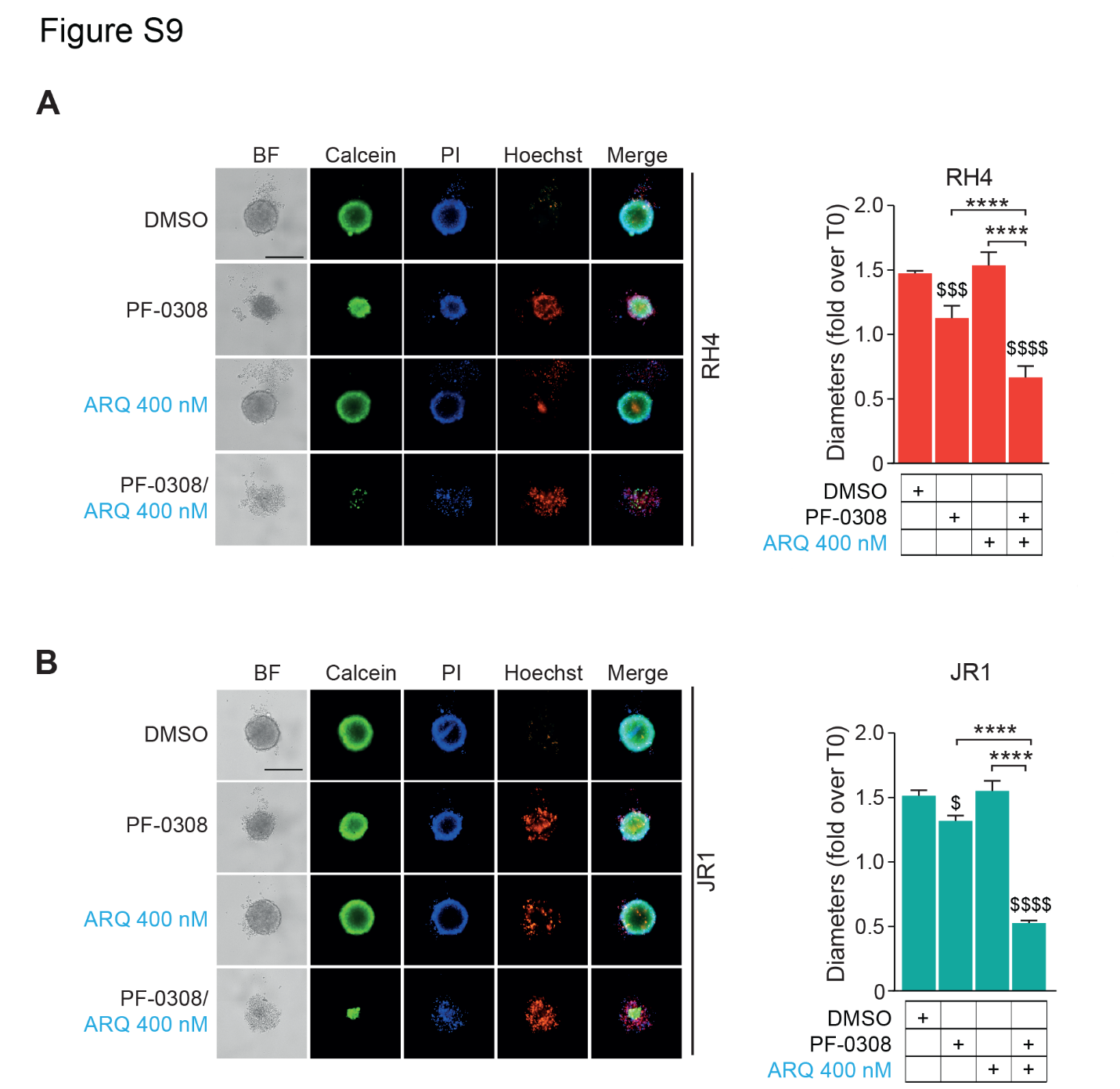
****** P-value ≤ 0.0001 for drug combination-treated cells *vs* single agents.

**Supplementary Figure 9. NOTCH and MET signaling co-inhibition induces growth arrest and cell death in RMS tumor spheroids. (A) (left)** Representative images of RH4 tumor spheroids treated for 72h with PF-03084014 (20 μM) and ARQ197 (400 nM), alone or in combination. Scale bars = 500 μm. **(right)** Histogram of spheroids’ diameters quantification in RH4 cells. Graphs represent the mean of three independent experiments ± SD, 2-way ANOVA test. $$$ P-value ≤ 0.001, $$$$ P-value ≤ 0.0001 for drug-treated cells *vs* vehicle-treated (DMSO) cells and **** P-value ≤ 0.0001 for drug combination-treated cells *vs* single agents. **(B)** **(left)** Representative images of JR1 tumor spheroids treated for 72h with PF-03084014 (10 μM) and ARQ197 (400 nM), alone or in combination. Scale bars = 500 μm. **(right)** Histogram of spheroids’ diameters quantification in JR1 cells. Graphs represent the mean of three independent experiments ± SD, 2-way ANOVA test. $ P-value ≤ 0.05, $$$$ P-value ≤ 0.0001 for drug-treated cells *vs* vehicle-treated (DMSO) cells and **** P-value ≤ 0.0001 for drug combination-treated cells *vs* single agents.
