# Supplementary material for: MET Inhibition Sensitizes Rhabdomyosarcoma Cells to NOTCH Signaling Suppression
Source: Front Oncol. 2022 Apr 27;12:835642. doi: 10.3389/fonc.2022.835642 (PMC9092259; doi:10.3389/fonc.2022.835642)
Supplement: Supplementary file 2 [file DataSheet_2.pdf]

MET Inhibition Sensitizes Rhabdomyosarcoma Cells to NOTCH Signaling Suppression

Clara Perrone [1,2#], Silvia Pomella [1#], Matteo Cassandri [1,3#],, Michele Pezzella [1§], Giuseppe Maria Milano[1§], Marta Colletti [1§], Cristina Cossetti [1] , Giulia Pericoli [1], Angela Di Giannatale [1], Emmanuel De Billy [1], Maria Vinci [1], Stefania Petrini [4], Francesco Marampon [3], Concetta Quintarelli [1], Riccardo Taulli [5], Josep Roma [6], Soledad Gallego [6], Simona Camero [7], Francesca Megiorni [8], Paolo Mariottini [2], Manuela Cervelli [2], Biagio De Angelis [1], Lucio Miele [9], Franco Locatelli [1,10], Rossella Rota [1],*

Uncropped western blots

Figure 1A

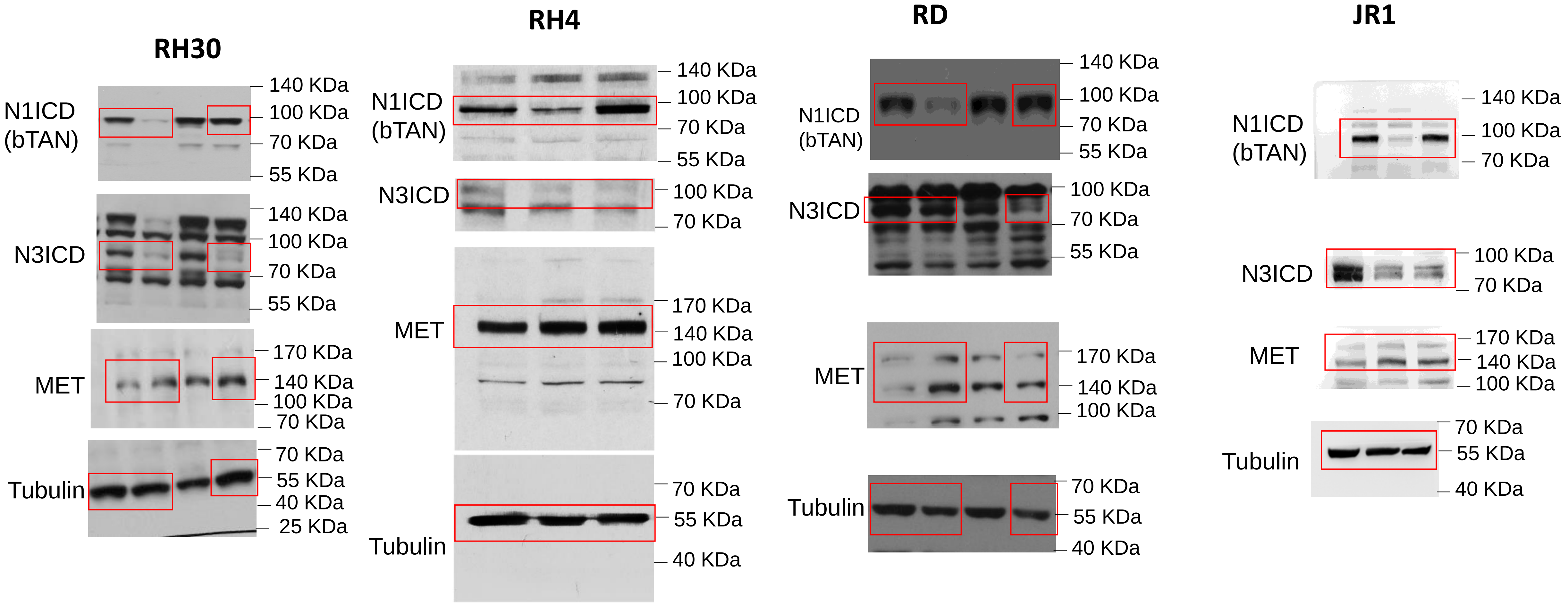
RH30
N1ICD (bTAN)
140 KDa
100 KDa
70 KDa
55 KDa
N3ICD
140 KDa
100 KDa
70 KDa
55 KDa
MET
170 KDa
140 KDa
100 KDa
70 KDa
Tubulin
70 KDa
55 KDa
40 KDa
25 KDa
RH4
N1ICD (bTAN)
140 KDa
100 KDa
70 KDa
55 KDa
N3ICD
100 KDa
70 KDa
MET
170 KDa
140 KDa
100 KDa
70 KDa
Tubulin
70 KDa
55 KDa
40 KDa
RD
N1ICD (bTAN)
140 KDa
100 KDa
70 KDa
55 KDa
N3ICD
100 KDa
70 KDa
55 KDa
MET
170 KDa
140 KDa
100 KDa
Tubulin
70 KDa
55 KDa
40 KDa
JR1
N1ICD (bTAN)
140 KDa
100 KDa
70 KDa
N3ICD
100 KDa
70 KDa
MET
170 KDa
140 KDa
100 KDa
Tubulin
70 KDa
55 KDa
40 KDa

Figure 1C

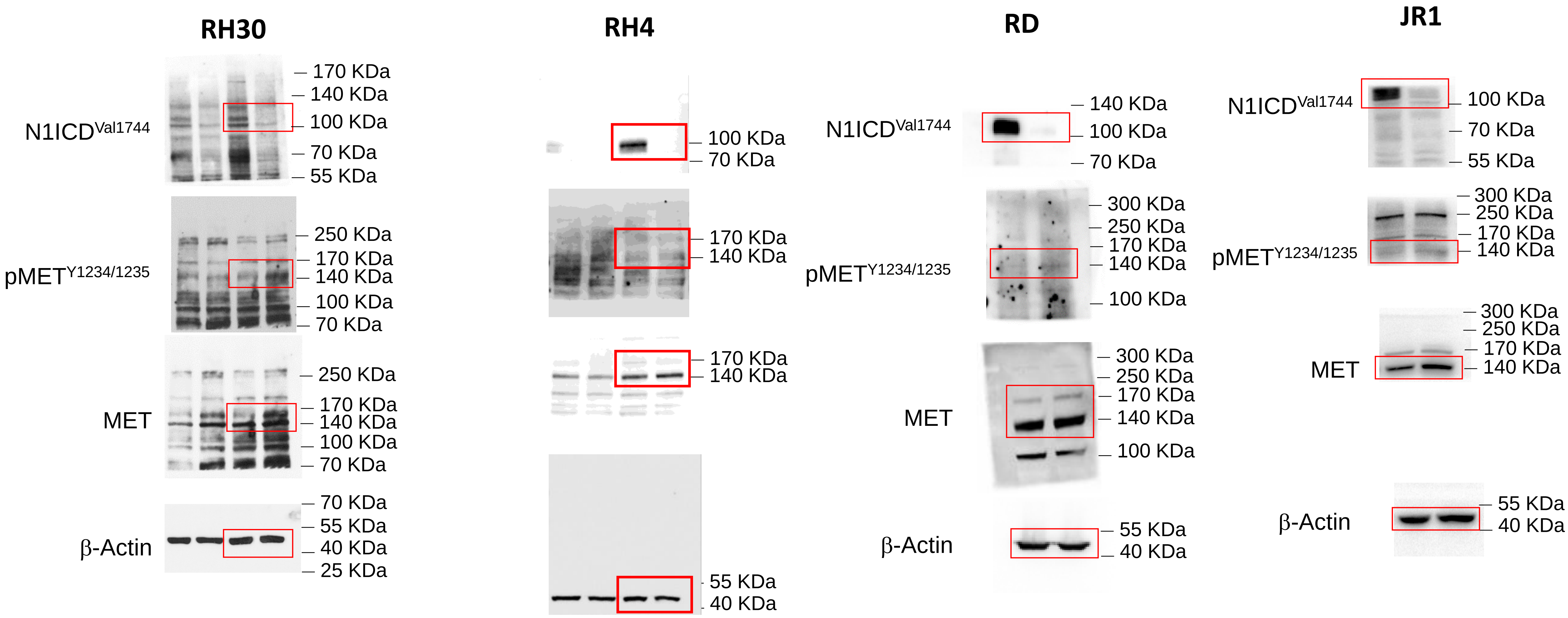
RH30
RH4
RD
JR1
N1ICDVal1744
pMETY1234/1235
MET
β-Actin
170 KDa
140 KDa
100 KDa
70 KDa
55 KDa
250 KDa
300 KDa
40 KDa
25 KDa

Figure 2B

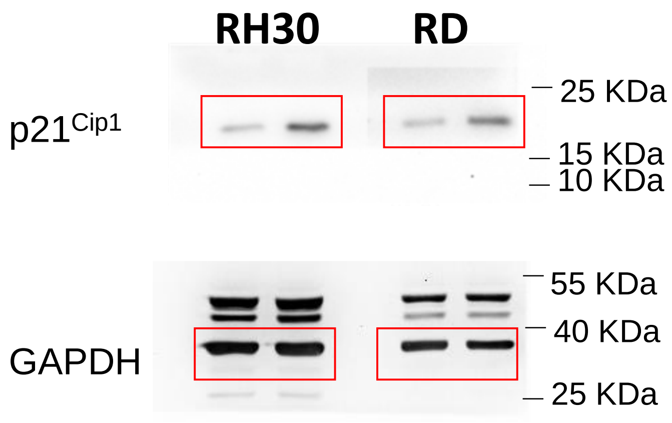

Figure 3A

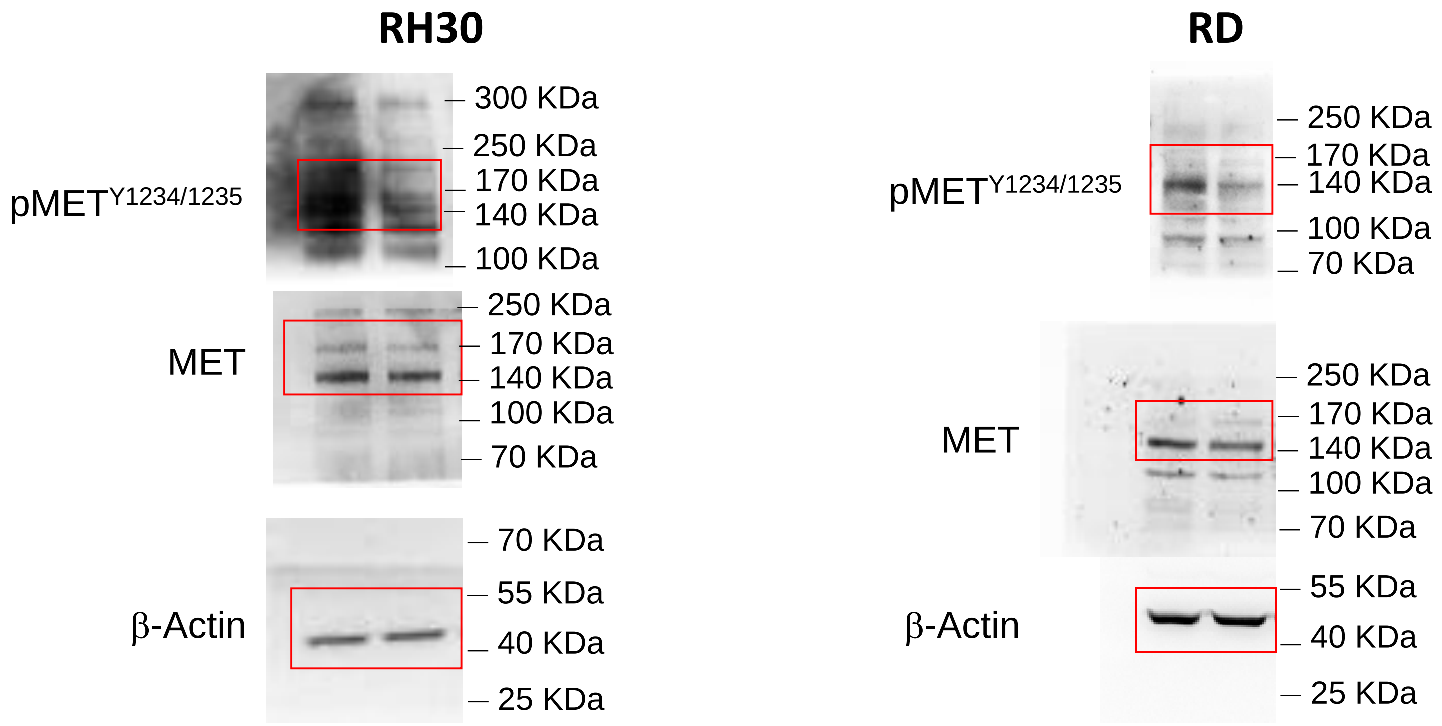

RH30
pMET$^{Y1234/1235}$
300 KDa
250 KDa
170 KDa
140 KDa
100 KDa
MET
250 KDa
170 KDa
140 KDa
100 KDa
70 KDa
β-Actin
70 KDa
55 KDa
40 KDa
25 KDa
RD
pMET$^{Y1234/1235}$
250 KDa
170 KDa
140 KDa
100 KDa
70 KDa
MET
250 KDa
170 KDa
140 KDa
100 KDa
70 KDa
β-Actin
55 KDa
40 KDa
25 KDa

Figure 3C

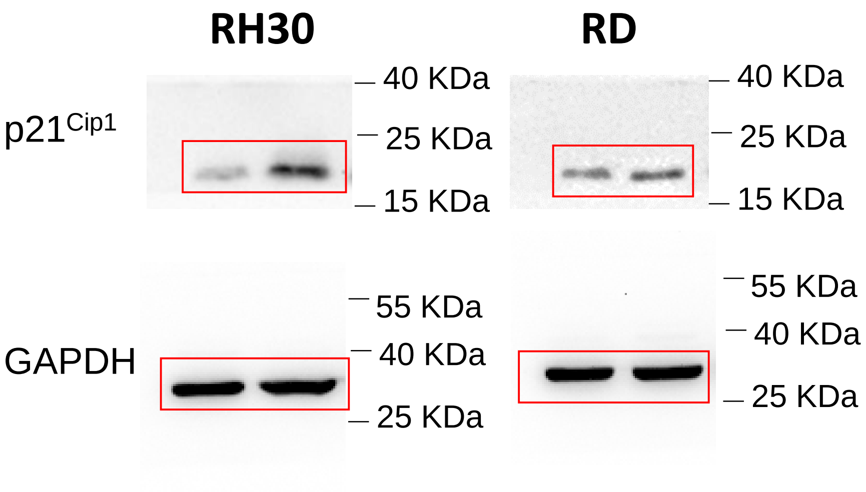

RH30
RD
p21Cip1
40 KDa
25 KDa
15 KDa
40 KDa
25 KDa
15 KDa
GAPDH
55 KDa
40 KDa
25 KDa
55 KDa
40 KDa
25 KDa

Figure 5D

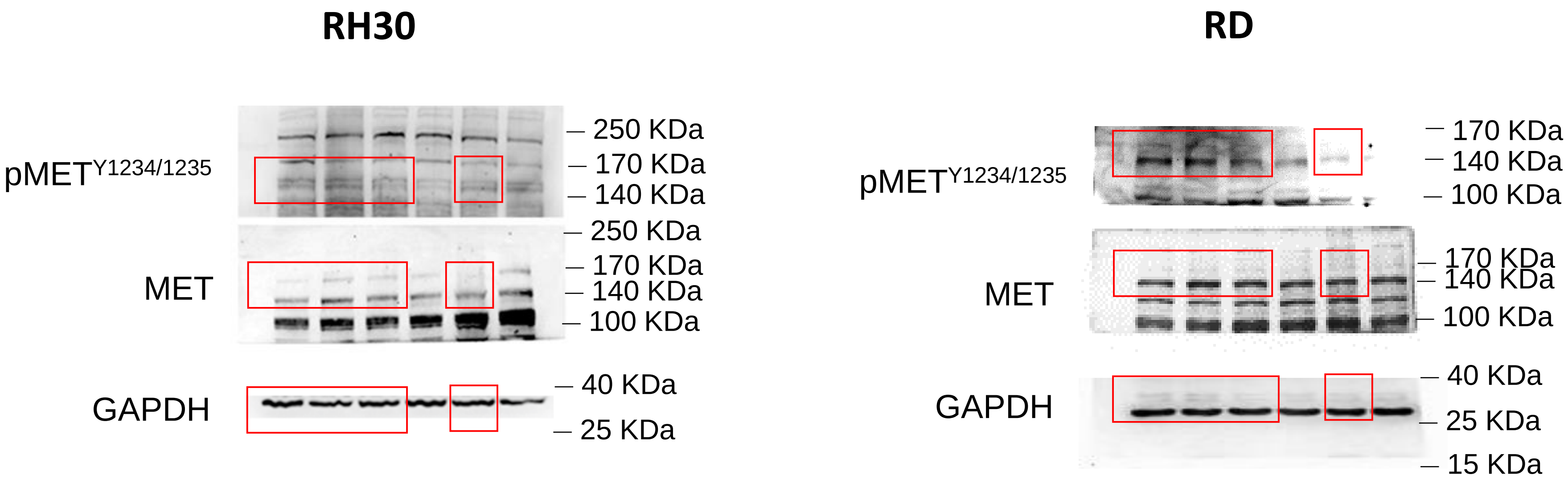

RH30
pMET$^{Y1234/1235}$
250 KDa
170 KDa
140 KDa
MET
250 KDa
170 KDa
140 KDa
100 KDa
GAPDH
40 KDa
25 KDa
RD
pMET$^{Y1234/1235}$
170 KDa
140 KDa
100 KDa
MET
170 KDa
140 KDa
100 KDa
GAPDH
40 KDa
25 KDa
15 KDa

Figure 5E

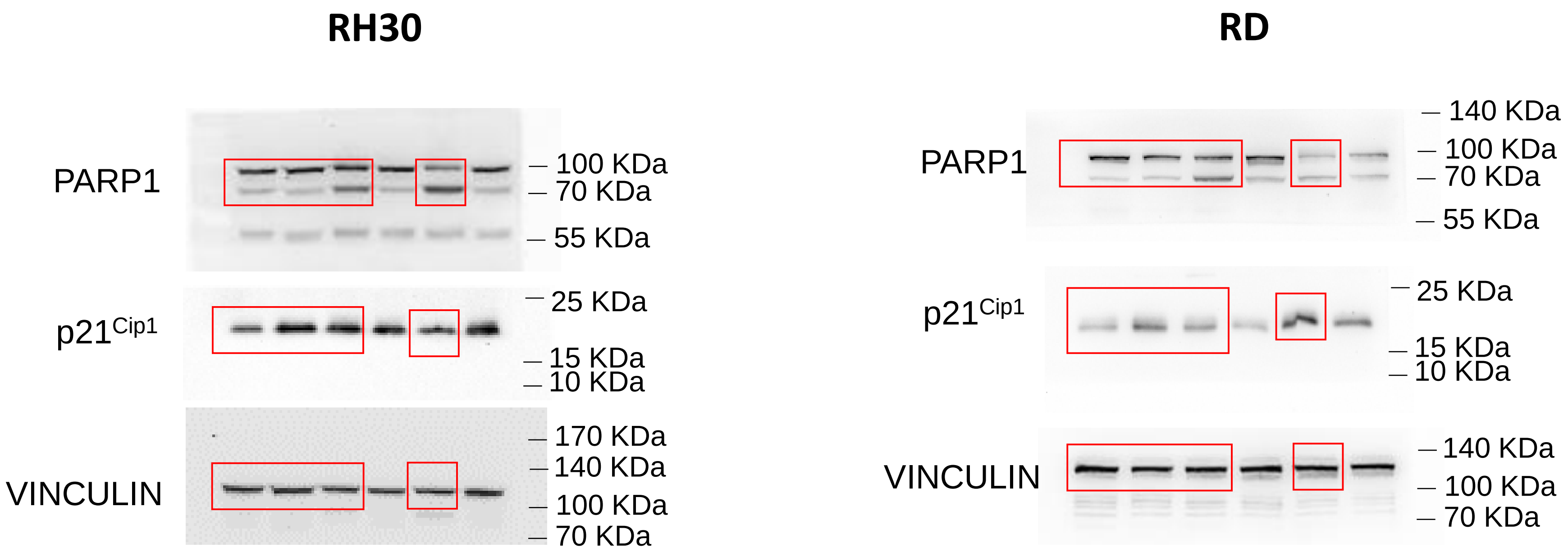
RH30
PARP1
100 KDa
70 KDa
55 KDa
p21Cip1
25 KDa
15 KDa
10 KDa
VINCULIN
170 KDa
140 KDa
100 KDa
70 KDa
RD
PARP1
140 KDa
100 KDa
70 KDa
55 KDa
p21Cip1
25 KDa
15 KDa
10 KDa
VINCULIN
140 KDa
100 KDa
70 KDa

Figure S2B

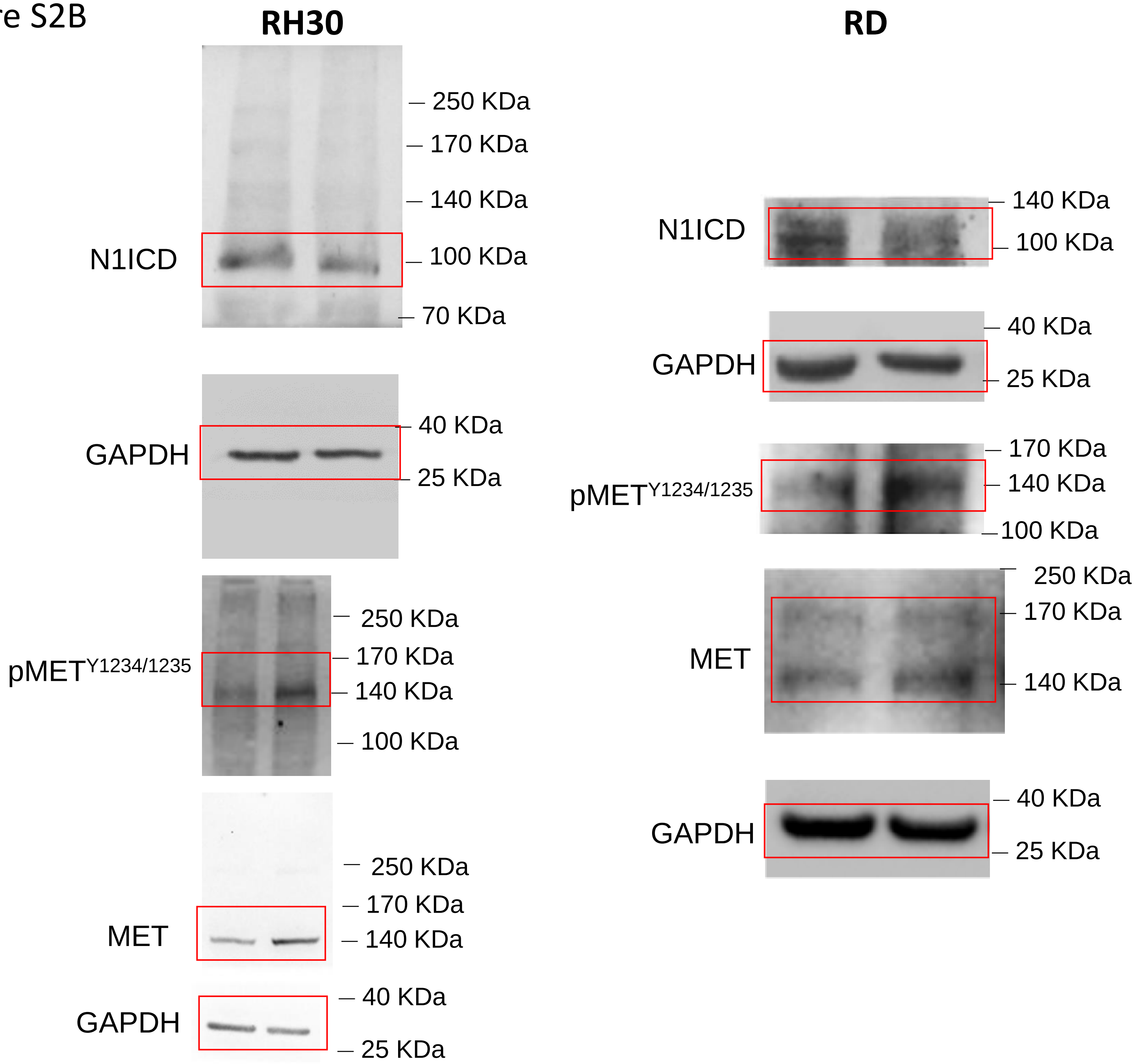
RH30
RD
N1ICD
250 KDa
170 KDa
140 KDa
100 KDa
70 KDa
GAPDH
40 KDa
25 KDa
pMET$^{Y1234/1235}$
MET

Figure S7D

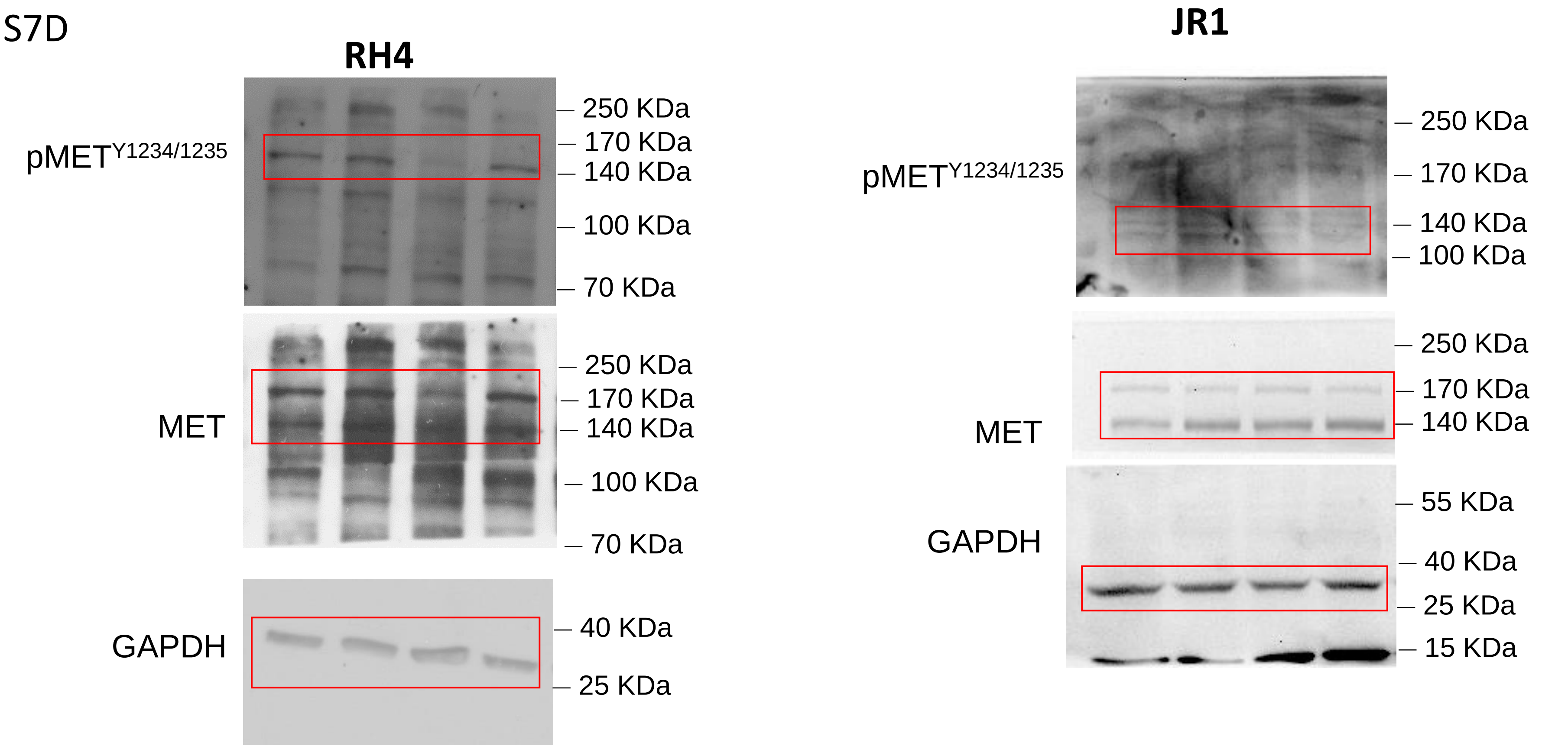

RH4
pMET Y1234/1235
250 KDa
170 KDa
140 KDa
100 KDa
70 KDa
MET
250 KDa
170 KDa
140 KDa
100 KDa
70 KDa
GAPDH
40 KDa
25 KDa
JR1
pMET Y1234/1235
250 KDa
170 KDa
140 KDa
100 KDa
MET
250 KDa
170 KDa
140 KDa
GAPDH
55 KDa
40 KDa
25 KDa
15 KDa

Figure S7E

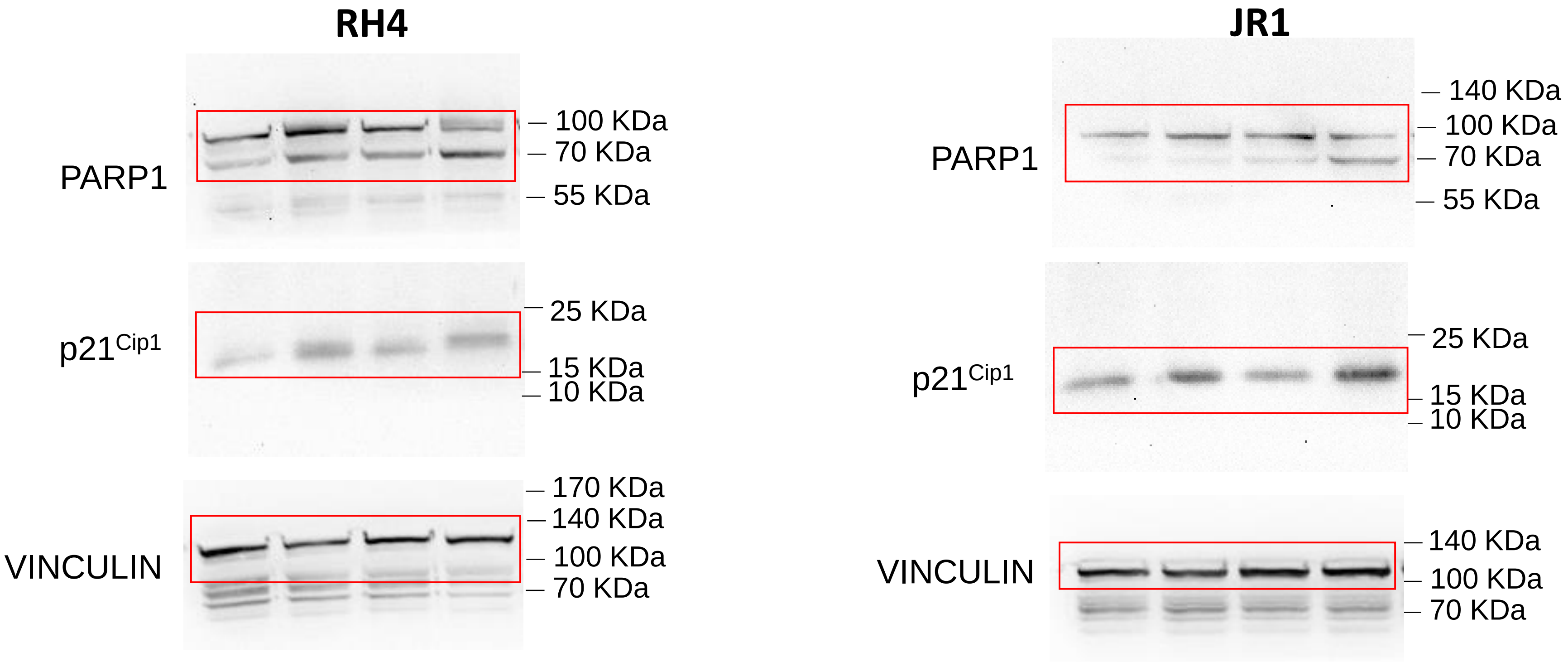
RH4
JR1
PARP1
p21Cip1
VINCULIN
100 KDa
70 KDa
55 KDa
25 KDa
15 KDa
10 KDa
170 KDa
140 KDa
100 KDa
70 KDa
140 KDa
100 KDa
70 KDa
55 KDa
PARP1
p21Cip1
VINCULIN
25 KDa
15 KDa
10 KDa
140 KDa
100 KDa
70 KDa
